# The impact of antidiabetic treatment on human hypothalamic infundibular neurons and microglia

**DOI:** 10.1172/jci.insight.133868

**Published:** 2020-08-20

**Authors:** Martin J.T. Kalsbeek, Samantha E.C. Wolff, Nikita L. Korpel, Susanne E. la Fleur, Johannes A. Romijn, Eric Fliers, Andries Kalsbeek, Dick F. Swaab, Inge Huitinga, Elly M. Hol, Chun-Xia Yi

**Affiliations:** 1 Laboratory of Endocrinology, and; 2 Department of Endocrinology and Metabolism, Amsterdam University Medical Center (UMC), University of Amsterdam, Amsterdam, Netherlands.; 3 Netherlands Institute for Neuroscience, an Institute of the Royal Netherlands Academy of Arts and Sciences, Amsterdam, Netherlands.; 4 Department of Medicine, Amsterdam UMC, University of Amsterdam, Amsterdam, Netherlands.; 5 Department of Translational Neuroscience, University Medical Center Utrecht Brain Center, Utrecht University, Utrecht, Netherlands.

**Keywords:** Endocrinology, Neuroscience, Diabetes, Insulin, NPY

## Abstract

Animal studies indicate that hypothalamic dysfunction plays a major role in type 2 diabetes mellitus (T2DM) development, and that insulin resistance and inflammation are important mechanisms involved in this disorder. However, it remains unclear how T2DM and antidiabetic treatments affect the human hypothalamus. Here, we characterized the proopiomelanocortin (POMC) immunoreactive (-ir) neurons, the neuropeptide-Y–ir (NPY-ir) neurons, the ionized calcium-binding adapter molecule 1–ir (iba1-ir) microglia, and the transmembrane protein 119–ir (TMEM119-ir) microglia in the infundibular nucleus (IFN) of human postmortem hypothalamus of 32 T2DM subjects with different antidiabetic treatments and 17 matched nondiabetic control subjects. Compared with matched control subjects, T2DM subjects showed a decrease in the number of POMC-ir neurons, but no changes in NPY-ir neurons or microglia. Interestingly, T2DM subjects treated with the antidiabetic drug metformin had fewer NPY-ir neurons and microglia than T2DM subjects not treated with metformin. We found that the number of microglia correlated with the number of NPY-ir neurons, but only in T2DM subjects. These results indicate that different changes in POMC and NPY neurons and microglial cells in the IFN accompany T2DM. In addition, T2DM treatment modality is associated with highly selective changes in hypothalamic neurons and microglial cells.

## Abstract 2

Type 2 diabetes impacts energy homeostasis-regulatory neurons and the surrounding microglia in the hypothalamus, and these effects are modulated by metformin.

## Graphical abstract

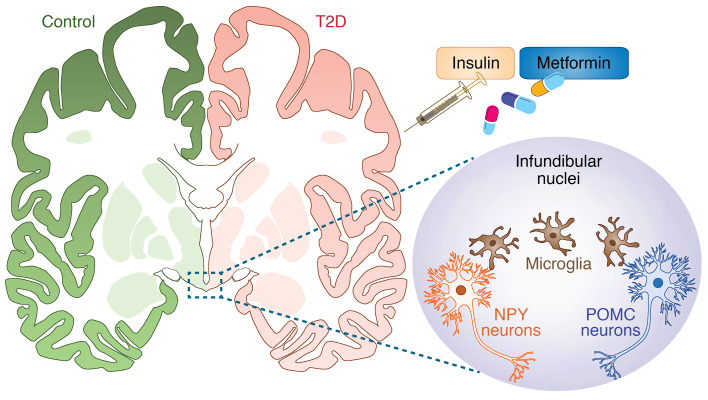

## Introduction

Hypothalamic dysfunction plays a major role in the development of obesity and type 2 diabetes (T2DM) (1, 2). Numerous studies have focused on the hypothalamic infundibular nucleus (IFN, equivalent to the arcuate nucleus in rodents), which contains 2 key neuronal populations regulating energy homeostasis, proopiomelanocortin-expressing (POMC-expressing) and neuropeptide Y (NPY)/agouti-related protein–expressing (AgRP-expressing) neurons. Diet-induced obesity and T2DM have been associated with a disturbed balance between the anorexigenic POMC (3, 4) and the orexigenic NPY/AgRP neurons (5, 6).

Recent animal studies have indicated hypothalamic inflammation as an important player in the changes in hypothalamic neuronal function observed during the development of obesity and T2DM (7–10). In these studies, hypothalamic inflammation was demonstrated by increased expression of proinflammatory factors mainly produced by reactive microglia, the resident innate immune cells of the brain. Although interactions between hypothalamic neurons and microglia are apparent in animal models (11–13), the clinical relevance of these findings in the pathophysiology of human T2DM remains unknown.

The diagnosis of T2DM in humans is based on elevated fasting plasma glucose levels. Antidiabetic treatments, which aim to lower glucose levels, entail a stepwise approach consisting of lifestyle changes and sequential addition of oral antidiabetics (standardly metformin) and insulin if necessary (14). The insulin sensitizer metformin lowers blood glucose levels via suppressing hepatic glucose production and increasing skeletal muscle glucose clearance (15). Metformin crosses the blood-brain barrier, affecting hypothalamic neuropeptide expression in diabetic rats (16) and decreasing NPY gene expression in cultured hypothalamic neurons (17). Likewise, insulin affects neuropeptide expression in the rodent hypothalamus (18, 19). Interestingly, in addition to their effect on neurons, both metformin and insulin have antiinflammatory properties (20, 21). These data indicate that oral antidiabetic medications and insulin analogs may influence both hypothalamic neurons and hypothalamic microglia.

In the current study, we aimed to answer the following questions: Are NPY and POMC neuronal numbers altered in T2DM? Is soma size, as a functional measure of the structural integrity and viability of neurons, altered in T2DM? Is microglial activity, as reflected by soma number, size, and ramification in the IFN, associated with changes in NPY and POMC neurons in T2DM? Does antidiabetic treatment have an impact on microglia and/or NPY and POMC neurons? Here, we characterized neurons and microglia in the IFN of T2DM subjects, using human postmortem brain tissue, with special attention to the possible effects of antidiabetic treatments.

## Results

### Distribution of NPY-ir neurons in the IFN is not different between control and T2DM subjects.

**Figure 1:**
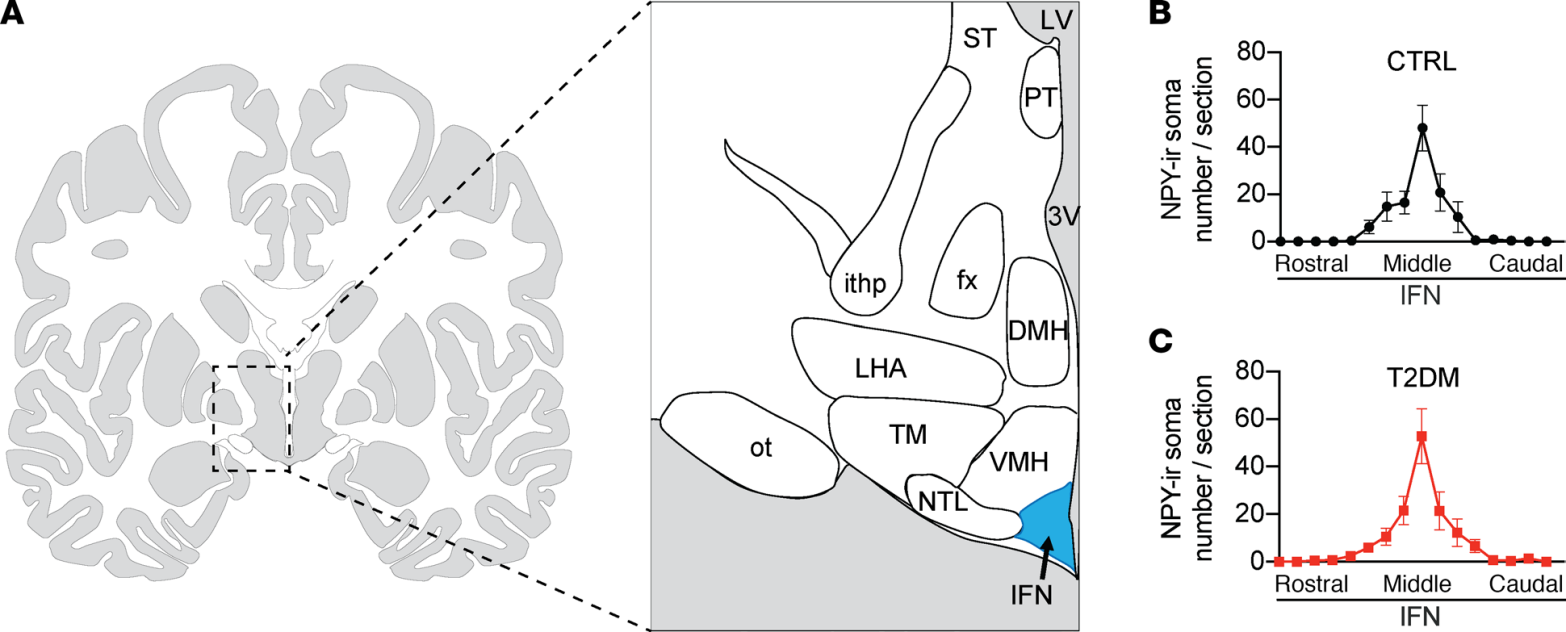
The section selection strategy. (**A**) Schematic of human hypothalamus showing the areas neuropeptide Y immunoreactivity (NPY-ir) neurons were counted in the infundibular nucleus (IFN) in the hypothalamus along the rostral-caudal axis. (**B** and **C**) The distribution pattern of NPY-ir neurons along the rostral to caudal axis in the IFN of the CTRL subjects (**B**) and the T2DM subjects (**C**) is similar. 3V, third ventricle; DMH, dorsomedial hypothalamus; fx, fornix; ithp, inferior thalamic peduncle; LV, lateral ventricle; NTL, lateral tuberal nucleus; LHA; lateral hypothalamus; ot, optic tract; PT, paratenial thalamic nucleus; ST, stria terminalis; TM, tuberomammillary hypothalamic nucleus; VMH, ventromedial hypothalamus.

The brain size of individual subjects and, consequently, the sagittal length of the hypothalamus varies considerably between subjects. To compare neuronal and glial parameters within the IFN and prevent between-group differences of subjects owing to brain size, we used the sections around the area in the IFN that showed the highest NPY immunoreactivity (-ir) to study the different neuron and microglial markers (Figure 1A). First, sequential sections approximately 100 sections apart, along the rostro-caudal axis, starting from the optic chiasm to the mammillary body of the hypothalamus, were visualized with NPY-ir. NPY-ir neurons are mainly present in the medial and central area of the IFN (Supplemental Figure 1A; supplemental material available online with this article; https://doi.org/10.1172/jci.insight.133868DS1). Plots of the total number of NPY-ir somas in every 100th section resulted in similar graphs for control (CTRL) (Figure 1B) and T2DM (Figure 1C) subjects. There was no significant difference in area under the curve between T2DM subjects and the matched CTRL subjects (*P* = 0.96), indicating the shape, size, and orientation of the IFN were comparable between the 2 groups.

### Metformin treatment is associated with decreased NPY-ir in the IFN, independent of additional insulin treatment.

**Figure 2:**
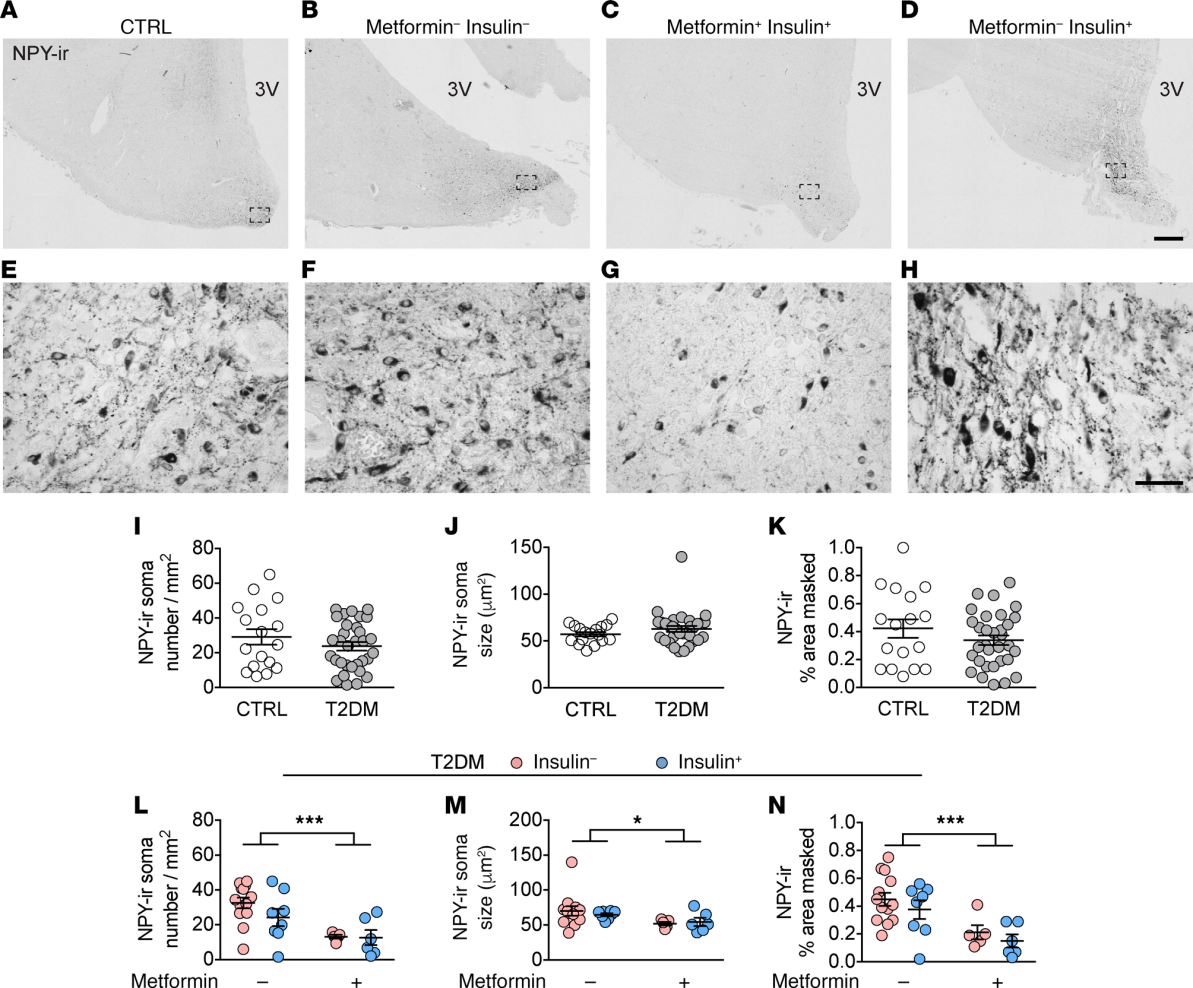
Metformin treatment in T2DM subjects is associated with decreased NPY-ir in the IFN, independent of additional insulin treatment. (**A**–**K**) NPY-ir neurons in the IFN of a CTRL subject (**A**), a T2DM subject with no insulin no metformin (**B**), with metformin and insulin (**C**), or with insulin (**D**) (higher magnification of the areas framed in **A**–**D** are shown in **E**–**H**, respectively). (**I**–**K**) Comparison between CTRL and T2DM subjects on: NPY-ir neuronal number (**I**), NPY-ir soma size (**J**), and the relative area covered by NPY-ir (**K**). (**L**–**N**) The antidiabetic treatment-associated effect on NPY-ir neuronal number (**L**), NPY-ir soma size (**M**), and the relative area covered by NPY-ir (**N**). 3V, third ventricle. Scale bars: 500 μm (**D**), 50 μm (**H**). Data represent mean ± SEM. Significance is calculated using 2-way ANOVA (**L**–**N**). All *P* values were corrected for multiple testing using the Benjamini-Hochberg criterion. *P < 0.05, ***P < 0.001.

We further characterized NPY-ir neurons in the central part of the IFN, measuring neuron number, soma size, and relative area covered by NPY-ir in both CTRL and T2DM subjects, with T2DM subjects further grouped by the antidiabetic treatments they had received (Supplemental Figure 2 and Figure 2, A–H). In CTRL subjects, BMI negatively correlated with the number of NPY-ir neurons and with the relative area covered by NPY-ir (Supplemental Figure 3). Notably, in the T2DM subjects with a matching BMI (Table 1), this correlation was absent for all NPY parameters (Supplemental Figure 4), which might indicate that the possible negative feedback of adiposity on NPY neurons in the CTRL subjects was lost in the T2DM subjects. Further, none of the other confounding factors (HbA1c, postabsorptive glucose, age, postmortem delay [PMD], fixation time, and brain weight) correlated to the NPY parameters in T2DM subjects (Supplemental Figure 4). In CTRL subjects, postabsorptive glucose and HbA1c data were limited. Therefore, we only analyzed the remaining confounding factors and found no significant correlations (Supplemental Figure 3). Considering that PMD was not correlated to NPY-ir soma number and size, we concluded that the difference in PMD between the CTRL and T2DM groups had no effect on our results.

We found no differences in NPY neuron number, soma size, and relative area of coverage between the T2DM and CTRL groups (Figure 2, I–K). We then analyzed the effects of antidiabetic treatment within the T2DM subjects. All confounders matched well between treatment groups, except that those who received insulin treatment were younger than those who did not (Table 1). Because metformin and insulin have an inhibitory effect on appetite and hyperglycemia (19, 22), we wanted to determine if T2DM subjects had different levels of NPY expression based on their treatment. Indeed, we found metformin-associated effects on NPY expression: fewer neurons, smaller soma, and smaller relative area covered by NPY-ir (Figure 2, L–N, *P* = 0.001, *P* = 0.036, and *P* = 0.0007, respectively). We did not observe an insulin-associated effect on any of these NPY-ir parameters (Figure 2, L–N). The observed metformin-associated decrease of NPY-ir parameters indicates a general loss of neuronal function, and may indicate the mechanism through which metformin can reduce appetite (i.e., by regulating NPY activity).

### Decreased POMC-ir in the IFN in T2DM subjects can be prevented by insulin treatment.

**Figure 3:**
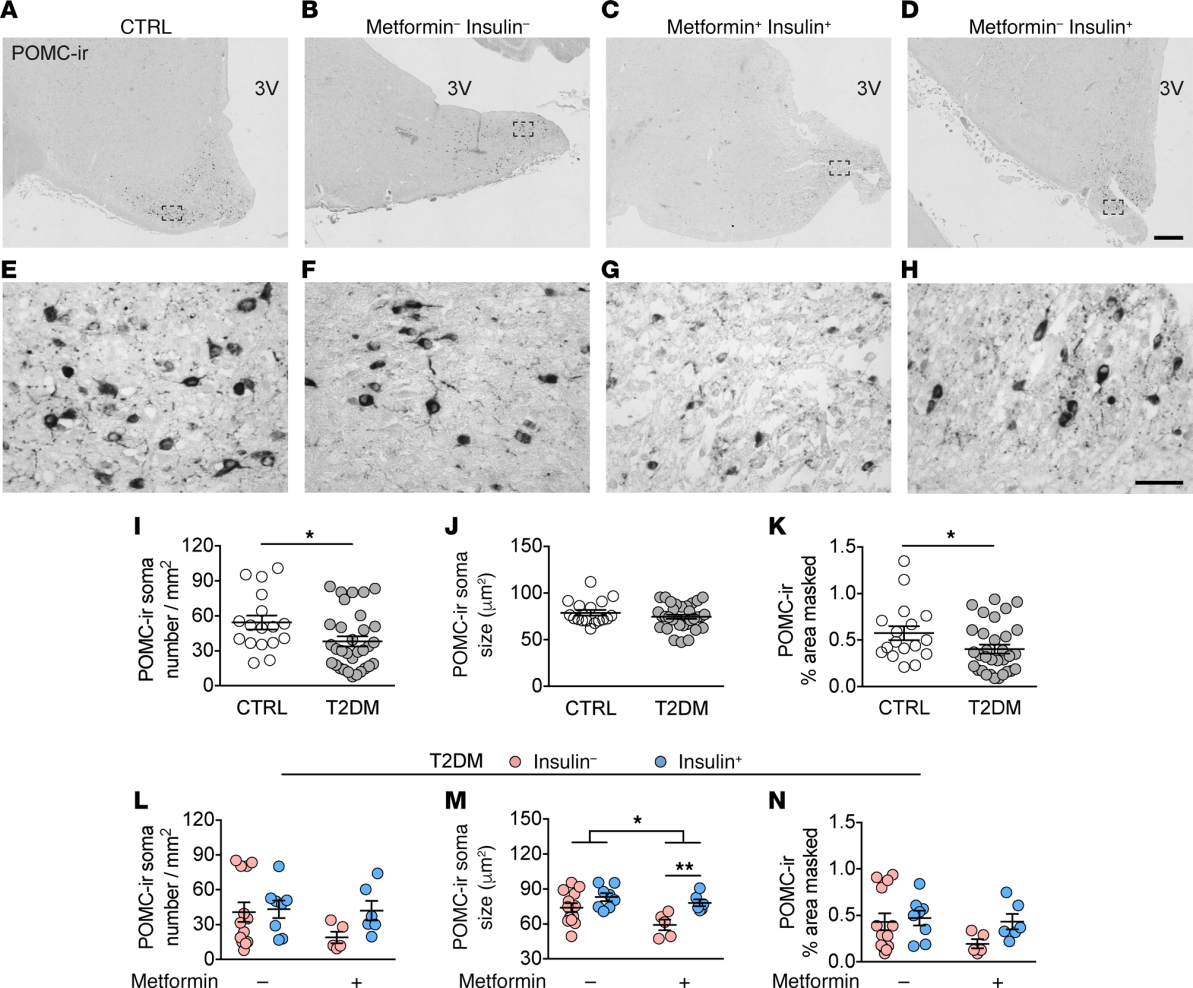
Decreased POMC-ir in the IFN of T2DM subjects can be prevented by insulin treatment. (**A**–**H**) POMC-ir neurons in the IFN of a CTRL subject (**A**), T2DM subject with no insulin no metformin (**B**), with metformin (**C**), or with insulin (**D**) (higher magnification of the areas framed in **A**–**D** are shown in **E**–**H**, respectively). (**I**–**K**) Comparison between CTRL and T2DM of: POMC-ir neuronal number (**I**), POMC-ir soma size (**J**), and the relative area covered by POMC-ir (**K**). (**L**–**N**) The antidiabetic treatment-associated effect on POMC-ir neuronal number (**L**), POMC-ir soma size (**M**), and the relative area covered by POMC-ir (**N**). 3V, third ventricle. Scale bars: 500 μm (**D**), 50 μm (**H**). Data represent mean ± SEM. Significance is calculated using an ordinary Student’s *t* test in **I**, a Mann-Whitney test in **K**, and a 2-way ANOVA in **M**. All *P* values were corrected for multiple testing using the Benjamini-Hochberg criterion. *P < 0.05, **P < 0.01.

Owing to the decrease of NPY-ir, we next evaluated whether changes occur in the other major neuronal population in the IFN, the POMC neurons. POMC staining in the central part of the IFN revealed that the POMC region largely overlapped with the NPY region, but extended into a wider more lateral and dorsal area (Supplemental Figure 1B and Figure 3, A–D). The staining was predominantly present in the cytoplasm of the cell bodies and less densely stained in fibers (Figure 3, E–H). Again, we checked for confounders. We found that in CTRL subjects, age negatively correlated with POMC-ir soma size and relative area covered by POMC-ir (Supplemental Figure 5), indicating a loss of POMC-ir cells owing to the aging process in general. However, because we matched the groups for age (Table 1), these correlations did not influence our conclusions on group effects. In T2DM subjects, age did not correlate to any POMC parameter (Supplemental Figure 6). Moreover, the other confounders did not correlate to any of the POMC-ir parameters in the CTRL subjects (Supplemental Figure 5), or in T2DM subjects (Supplemental Figure 6).

We found that, compared with the matched CTRL group, the POMC-ir neurons and the area covered by POMC-ir were significantly lower in the T2DM group (Figure 3, I and K, q = 0.04 and q = 0.0499, respectively), indicating a general decrease of POMC expression in T2DM subjects, although no difference in POMC-ir soma size was found (Figure 3J). Moreover, these data also suggest that the T2DM pathogenesis has a distinctive detrimental impact on the cell biology of NPY and POMC neurons.

As mentioned earlier, the only confounding factor between treatment groups was age, e.g., insulin-treated subjects died at a significantly younger age than T2DM subjects without insulin treatment (Table 1, *P* = 0.01). However, this did not influence our conclusions as the T2DM group age showed no confounding effect on the POMC-ir parameters (Supplemental Figure 6). The remaining confounding factors matched well between the T2DM groups (Table 1). We found contrasting effects associated with the antidiabetic treatments on POMC-ir neurons (Figure 3, L–N). In those treated with metformin, a combination with insulin treatment was associated with larger POMC-ir somas (Figure 3M, *P* = 0.003), whereas metformin treatment associated with smaller POMC-ir somas (Figure 3M, *P* = 0.031). T2DM subjects in general have decreased levels of POMC expression, which can be prevented by long-term insulin treatment. These results imply a diverse impact of insulin and metformin on NPY and POMC expression.

### T2DM subjects treated with metformin show a regional-specific decrease in the number of iba1-ir microglial soma and ramifications.

**Figure 4:**
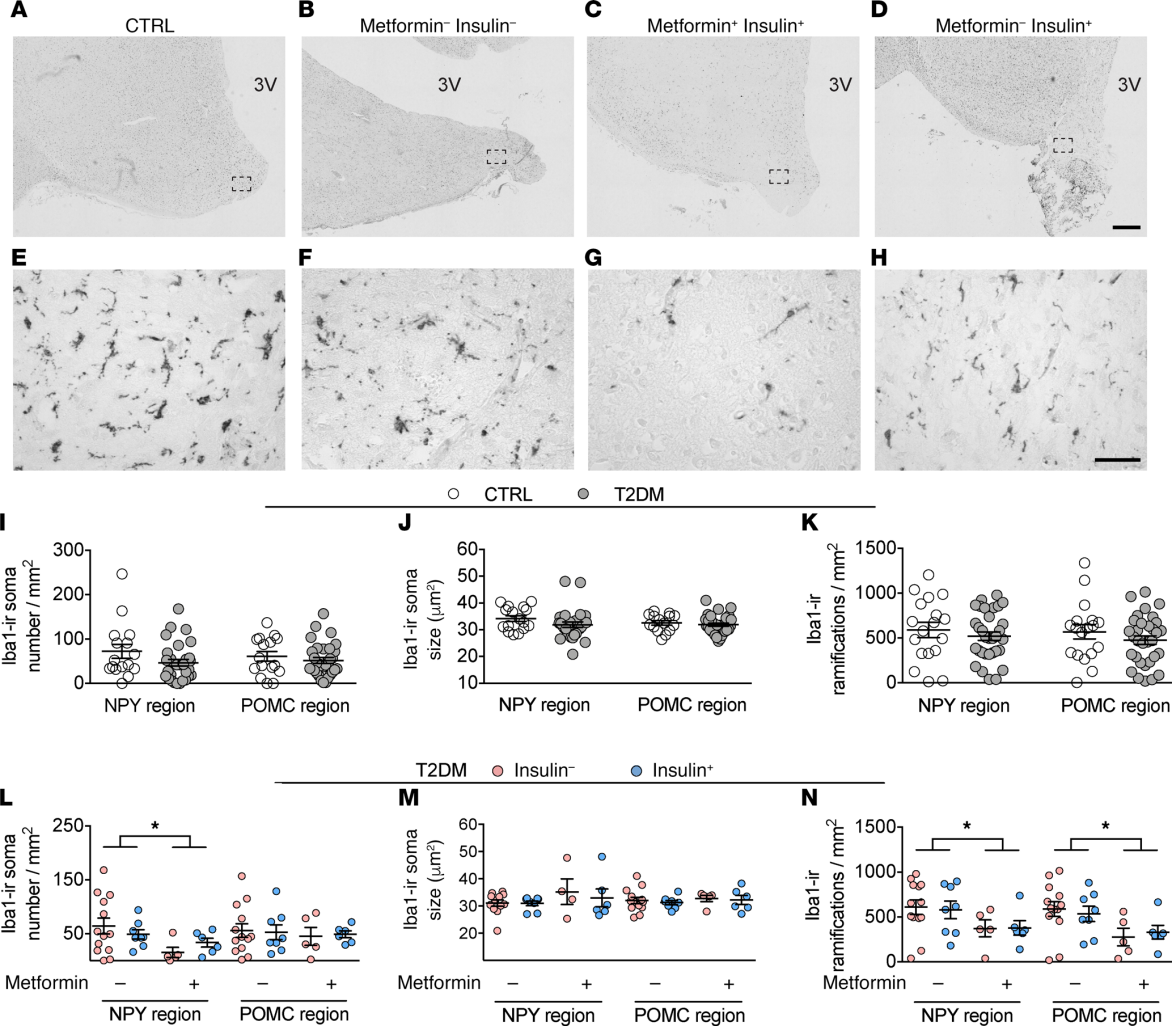
T2DM subjects treated with metformin show a region-specific decrease in the number of iba1-ir microglial soma and ramifications in the IFN. (**A**–**H**) iba1-ir microglia in the IFN of a CTRL subject (**A**), T2DM subject with no insulin no metformin (**B**), with metformin and insulin (**C**), or with insulin (**D**) (higher magnification of the areas framed in **A**–**D** are shown in **E**–**H**, respectively). (**I**–**K**) Comparison in the neuropeptide Y (NPY) and POMC region of: iba1-ir soma number (**I**), iba1-ir soma size (**J**), and number of iba1-ir ramifications (**K**). (**L**–**N**) Antidiabetic treatment-associated effects in the NPY and POMC region on iba1-ir soma number (**L**), iba1-ir soma size (**M**), and number of iba1-ir ramifications (**N**). 3V, third ventricle. Scale bars: 500 μm (**D**), 50 μm (**H**). Data are represented as mean ± SEM. Significance is calculated using 2-way ANOVA in **L** and **N**. All *P* values were corrected for multiple testing using the Benjamini-Hochberg criterion. *P < 0.05.

To investigate the microglial changes in the IFN of T2DM subjects, we profiled the microglia using ionized calcium-binding adapter molecule 1 (iba1), because it is the best structural marker for visualizing microglia in gray matter (23). The iba1-ir staining showed microglial cells throughout the IFN (Figure 4, A–D). The iba1 staining was present in the cell bodies as well as in the ramifications (Figure 4, E–H). The full morphology of individual iba1-ir cells was impossible to determine owing to the limited section thickness. Therefore, we quantified iba1-ir soma number and size as well as the number of ramifications. An increase of these parameters compared with normal microglia is an indication of higher microglial activity.

We investigated the same confounding factors as previously described and found that, in the NPY region of CTRL subjects, brain weight positively correlated to the number of iba1-ir soma and ramifications (Supplemental Figure 7). A previous study reported a smaller brain weight in association with a loss of myelination owing to the lack of insulin-like growth factor 1 (IGF1) in CD11c (mainly expressed by macrophages and to a lesser extent by microglia) positive cells (24). The role that IGF1 in aged human brain microglia plays in maintaining myelination needs to be further studied. We also found that BMI positively correlated to iba1-ir soma size (Supplemental Figure 7), pointing to a possible association between obesity and microglial activity. However, our analysis on group differences was not affected by these 2 confounding factors because we matched all groups for both BMI and brain weight (Table 1). In the NPY region of T2DM subjects and the POMC region of CTRL and T2DM subjects, we found no correlation between iba1-ir and brain weight, BMI, postabsorptive glucose, and HbA1c (Supplemental Figures 8–10).

Because diet-induced obesity, prediabetes, and hypothalamic neuronal dysfunction are associated with increased microglial activity in animal studies, we expected an increase of microglia in our T2DM subjects as well. However, in the T2DM and CTRL subjects, we were unable to find any differences in iba1-ir parameters, in the NPY or POMC regions (Figure 4, I–K).

With the antiinflammatory properties of both metformin and insulin in mind, we anticipated a treatment- associated effect on microglial activity in these T2DM subjects. Surprisingly, we only found an effect associated with metformin treatment, and only in the NPY region (2-way ANOVA showed that metformin treatment associated with fewer iba1-ir cell bodies and fewer iba1-ir ramifications; Figure 4, L and N, *P* = 0.037, *P* = 0.012, respectively). We found no insulin-associated effect on any of the iba-1 parameters (Figure 4, L–N). Additionally, in the POMC region we were unable to locate any treatment-associated effects (Figure 4, L–N).

### Locally decreased TMEM119-ir in the NPY area in the IFN of the T2DM subjects treated with metformin.

**Figure 5:**
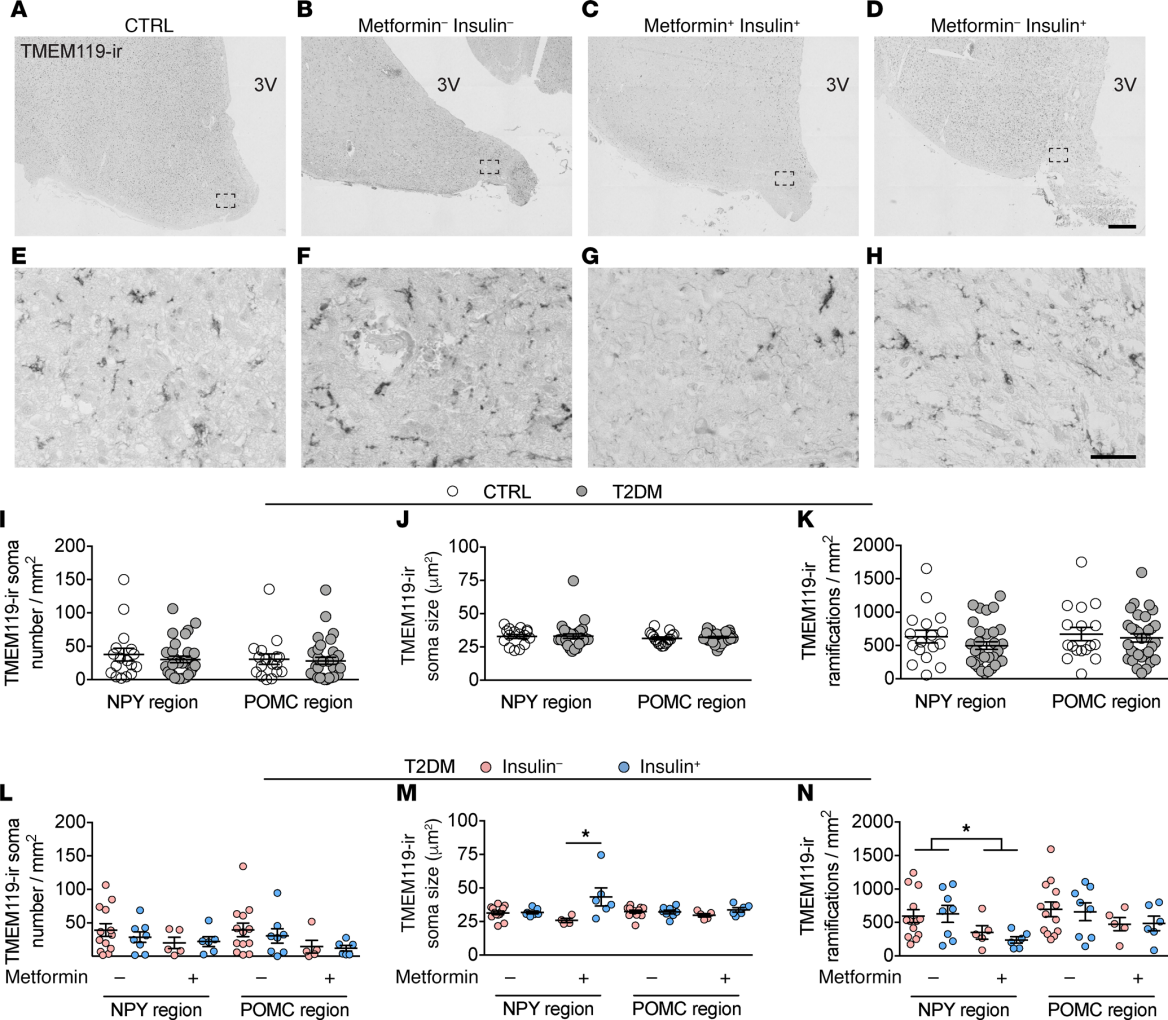
Locally changed TMEM119- ir in the NPY area in the IFN of the T2DM subjects treated with metformin. (**A**–**H**) TMEM119-ir microglia in the IFN of a CTRL subject (**A**), T2DM subject with no insulin no metformin (**B**), with metformin and insulin (**C**), or with insulin (**D**) (higher magnification of the areas framed in **A**–**D** are shown in **E**–**H**, respectively). (**I**–**K**) Comparison in the NPY and POMC region between CTRL and T2DM of: TMEM119-ir soma number (**I**), TMEM119-ir soma size (**J**), and the number of TMEM119-ir ramifications (**K**). (**L**–**N**) The antidiabetic treatment-associated effects in the NPY and POMC region on TMEM119-ir soma number (**L**), TMEM119-ir soma size (**M**), and the number of TMEM119-ir ramifications (**N**). 3V, third ventricle. Scale bars: 500 μm (**D**), 50 μm (**H**). Data are represented as mean ± SEM. Significance is calculated using 2-way ANOVA in **M** and **N**. All *P* values were corrected for multiple testing using the Benjamini-Hochberg criterion. *P < 0.05.

To gain more insight in the activation state of the microglia, we also profiled transmembrane protein 119 immunoreactivity (TMEM119-ir). This transmembrane protein negatively correlates with microglia activity and is primarily expressed by residential microglia (25, 26). TMEM119 showed a similar staining pattern of immunoreactivity as iba1, both present on the cell bodies as on the ramifications (Figure 5, A–H). Likewise, the full morphology of each microglia was impossible to determine because of the limited thickness of the sections.

In CTRL subjects, BMI had a significant impact on TMEM119-ir microglia, because it negatively correlated with the number of ramifications in both the NPY and POMC regions and negatively correlated with the number of somas in the POMC region (Supplemental Figures 11 and 12), again pointing to an association between obesity and microglial activity. Contrary to the iba1-ir results, the TMEM119-ir parameters did not correlate to brain weight (Supplemental Figures 11 and 12), which suggests that only infiltrating macrophages may be linked to brain atrophy, which seems consistent with the previously mentioned association between brain weight and lack of IGF1 in CD11c macrophages and/or microglia (24). In T2DM subjects, we found no further significant confounding in either the NPY or the POMC regions (Supplemental Figures 13 and 14).

The average number of TMEM119-ir microglia was roughly one-half the number of iba1-ir microglia (Figure 4 and Figure 5). Similar to the iba1-ir results, there were no differences in TMEM119-ir microglia between T2DM and CTRL subjects (Figure 5, I–K). We also found no treatment-associated effects in the POMC region (Figure 5, L–N). In the NPY region, however, there was a metformin-associated decrease in the number of TMEM119-ir ramifications (Figure 5N, *P* = 0.011). In addition, the combined effect of insulin plus metformin treatment increased TMEM119-ir microglial soma size in the NPY region (Figure 5M, *P* = 0.011).

**Figure 6:**
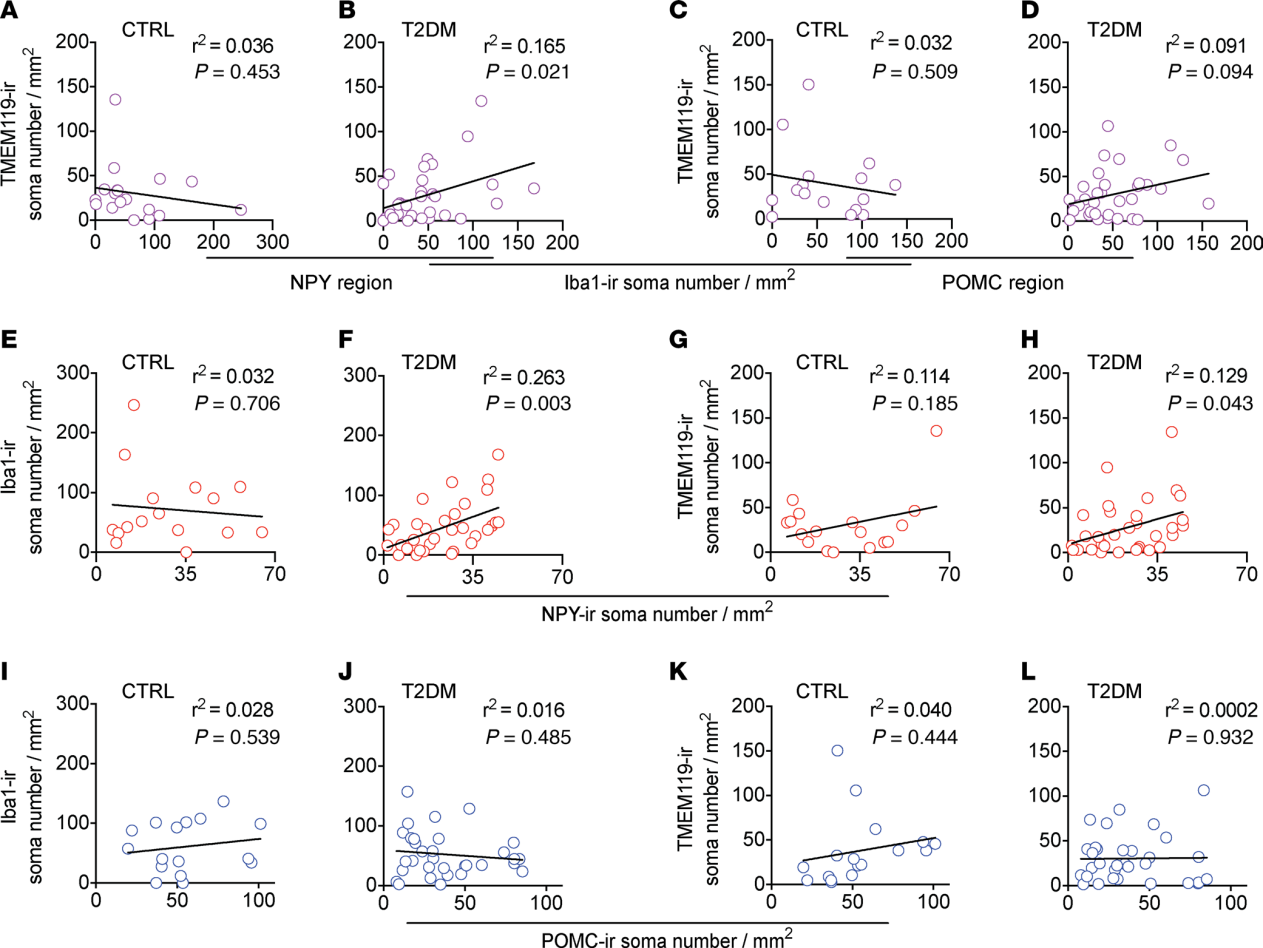
The association between the iba1-ir microglia and the TMEM119-ir microglia and the association between the number of neurons and the number of microglia in the IFN. (**A** and **B**) Plots of the number of iba1-ir microglia with the number of TMEM119-ir microglia in the NPY region of CTRL subjects (**A**) and T2DM subjects (**B**). (**C** and **D**) Plots of the number of iba1-ir microglia with the number of TMEM119-ir microglia in the POMC region of CTRL subjects (**C**) and T2DM subjects (**D**). (**E**–**H**) The number of NPY-ir neurons plotted against the number of iba1-ir microglia in CTRL subjects (**E**) and T2DM subjects (**F**), and against the number of TMEM119-ir microglia in CTRL subjects (**G**) and T2DM subjects (**H**). (**I**–**L**) The number of POMC-ir neurons plotted against the number of iba1-ir microglia in CTRL subjects (**I**) and T2DM subjects (**J**), and against the number of TMEM119-ir microglia in CTRL subjects (**K**) and T2DM subjects (**L**).

The relationship between iba1-ir and TMEM119-ir cells may shed some light on the regulation of resident microglia (expressing TMEM119-ir and iba1-ir) and infiltrating macrophages (expressing iba1-ir, but less TMEM119-ir) in the IFN. Therefore, we examined the correlation between these 2 markers. Intriguingly, in the NPY region of CTRL subjects, we found no correlation between the number of iba1 and TMEM119-expressing cells (Figure 6A), which suggests either an increased activity of microglia or an increase of nonresidential microglia, likely invading macrophages. In contrast, in the NPY region of T2DM subjects, iba1-ir and TMEM119-ir showed a significant correlation (Figure 6B), indicating that changes in the number of total immune cells are partly the result of a changed number of residential microglia. In the POMC region, we found no correlation between the number of iba1-ir and TMEM119-ir cells, in either the CTRL or T2DM subjects (Figure 6, C and D), again suggesting a higher presence of invaded macrophages in this region, compared with the NPY region of T2DM subjects.

### Number of microglia are positively correlated to number of NPY-ir neurons.

The local decrease of microglia in the NPY region raised the question about whether this correlated to the number of neurons present in this region. Therefore, we next investigated if there was a correlation between the number of neurons and microglia in the 2 different regions.

In CTRL subjects, the number of NPY-ir neurons did not correlate to the number of iba1-ir cells in that region (Figure 6E).However, in T2DM subjects, the number of NPY-ir neurons positively correlated to the number of iba1-ir cells (Figure 6F, *P* = 0.003). Additionally, in CTRL subjects we found no correlation between NPY-ir neurons and TMEM119 microglia (Figure 6G); however, we found a positive correlation in T2DM subjects (Figure 6H, *P* = 0.043). Contrary to the NPY-ir neurons, the number of POMC-ir neurons did not correlate with the number of iba1-ir or TMEM119-ir microglia in CTRL or T2DM subjects (Figure 6, I–L). Together, these results suggest that in T2DM, there are more interactions between NPY neurons and microglia/macrophages, whereas the lack of interaction between POMC-ir neurons and microglia is likely responsible for the loss of POMC-ir neurons in the T2DM.

## Discussion

This is the first study to our knowledge that examines the relationship between different antidiabetic treatments of T2DM patients and alterations in neurons and microglia in the human hypothalamus. We found clear differences in POMC-ir, NPY-ir, iba1-ir, and TMEM119-ir cell numbers and cell sizes in T2DM subjects, especially when taking into account their antidiabetic treatments. POMC-ir decreased in the T2DM group as a whole, but this decrease was not observed in subjects who had received insulin treatment. In contrast, NPY-ir and iba1-ir/TMEM119-ir in T2DM subjects were not different compared with CTRL subjects. However, within the T2DM subjects, NPY-ir was significantly lower in T2DM subjects treated with metformin. Moreover, iba1-ir and TMEM119-ir also decreased in T2DM subjects who had received metformin treatment, exclusively in the NPY region. Finally, T2DM individuals without metformin treatment had larger NPY-ir neuronal soma than CTRL subjects.

The role of the hypothalamic cells in the pathophysiology of T2DM has been studied extensively in animal models. However, studies in the hypothalamus of human T2DM patients are scarce, and the few studies performed to date show conflicting results. One study showed an increase of NPY-ir neurons without changes in the POMC-ir neurons (6), whereas a different study showed no effect on NPY-ir neurons, but a decrease of POMC-ir neurons (3). Both studies used postmortem brain tissue and matched the T2DM and CTRL subjects for sex, age, and PMD. Because only one study reported the BMI of the subjects, this may explain the conflicting results, although the same study showed there is no correlation between BMI and number of NPY-ir or POMC-ir neurons (3). In addition to possible (but unascertainable) differences in diet composition and genetic background, the one clear and major difference between these 2 studies is the antidiabetic treatment the subjects received. The T2DM subjects from Saderi et al. received “very limited to nil” treatment (6), whereas the T2DM subjects from Alkemade et al. all received treatment “with oral antidiabetics or insulin” (3). Thus, comparison of T2DM subjects with a different treatment history can lead to different results. The antidiabetic treatment itself might be accountable, or it could be a result of these patients being in different stages of the disease and requiring different treatments.

Metformin is widely used as the standard first line agent for treating T2DM. Consequently, those patients only on metformin treatment are in a different disease stage than those requiring insulin treatment. Metformin decreases intestinal glucose uptake, decreases hepatic glucose production, and increases insulin sensitivity (27–30). Furthermore, metformin inhibits food intake and reduces body weight gain in animals (17, 31, 32). Additionally, in humans metformin is known to decrease food consumption, body weight, and appetite (22, 33, 34). The exact mechanisms underlying these effects are unclear; however, it seems likely that the hypothalamus, which is the control center of energy homeostasis, is involved. Some studies have explored the relationship between metformin treatment and POMC and NPY expression. In diabetic rats, metformin inhibited the hypothalamic expression of NPY mRNA, but had no effect on POMC mRNA expression (17). In our study, we found a clear association of metformin treatment with a decrease of all NPY-ir parameters, indicating that in T2DM patients, long-term metformin treatment might be able to suppress NPY expression and its orexigenic effect. The lack of a correlation between NPY-ir and glycemic markers indicates that metformin does not affect NPY-ir through its glucose-lowering properties, but rather by a direct effect on NPY-ir neurons.

Previous animal studies show that hypothalamic inflammation is involved in disrupting the hypothalamic control of energy homeostasis, partly reflected by an increase of microglia number and size (4). The effect of metformin on microglial function is relatively unknown; however, results from animal studies generally show that metformin reduces microglial expression of proinflammatory cytokines and that this has a neuroprotective effect (35–38). Our study did not reveal differences in microglial markers between T2DM and CTRL subjects in general, but T2DM subjects with metformin treatment did have fewer microglial cell bodies and ramifications in the NPY region compared with controls. This is in contrast to the increase of microglia seen in the preceding animal studies. This discrepancy could be explained by the fact that these animals usually do not receive antidiabetic treatment as humans do. Another explanation could be that in animal studies the animals remain in a prediabetic stage and have not reached the actual diabetic phase, which would mean we are looking at different phases of the disease. Additionally, the microglia activation found in animals is already present within weeks after the start of the intervention (4), whereas our patient cohort consisted of relatively aged subjects, indicating our results reflect long-term effects of diabetes on microglia.

Interestingly, in addition to the positive correlations between the number of NPY-ir neurons and the number of iba1-ir/TMEM119-ir microglia in T2DM subjects, we also observed that the metformin-treated group had the lowest number of NPY-ir neurons. Thus, it seems unlikely that inhibition of microglia activity has a neuroprotective effect. Rather, it indicates that microglia activity may adapt to the neuronal demand for scavenging activity (i.e., less demand from neurons results in less microglial scavenging activity). Such correlation was only found between microglia and NPY neurons, suggesting that interaction between microglia and NPY neurons is substantially different from the interaction between microglia and the POMC neurons in T2DM. In our previous animal study, we found that on a high-fat diet reactive microglia moved closer to POMC neurons (12). This raises the possibility that in a metabolic disordered brain, POMC neurons are more vulnerable and have a higher demand for scavenging activity by microglia. However, in the long term, this microglia — POMC neuron interaction becomes defective or even detrimental, which results in POMC neuronal loss, as observed here. In contrast, NPY neurons may possess a stronger or more adequate survival mechanism. Different neuropeptides and neurotransmitters produced by NPY and POMC neurons may be underlying these specific neuron-microglial interactions. Microglia express receptors for many neurotransmitters, including NPY and α-MSH (39, 40). Moreover, chemical species such as ROS play important roles in the central regulation of energy metabolism that may differ between NPY and POMC neurons (41) and therefore result in a differential role of microglia in the vicinity of these neurons.

In conclusion, our study shows that T2DM pathology differently affects neuropeptide POMC and NPY expression in the hypothalamus in relation to antidiabetic treatment. Microglia are not significantly affected by T2DM pathology itself, but rather by the antidiabetic treatment.

## Methods

### Subjects information.

All brain materials were obtained from the Netherlands Brain Bank. The donors or their next of kin gave informed consent for a brain autopsy and for the use of the brain material and medical records for research purposes. Subjects who died with severe inflammation associated with brain tumor, encephalitis, or sepsis were excluded because of known confounders (42–44). Subjects diagnosed with an eating disorder or type 1 diabetes were also excluded. Subjects who had Braak stages V–VI (45) were excluded, as indications of severe dementia, although Alzheimer neuropathology is minimal in the IFN (46). When medical records did not report severe dementia, subjects lacking a Braak stage analysis were included as nondemented CTRL (Supplemental Table 1).

In total, 49 postmortem human hypothalamic samples were studied, 32 subjects were diagnosed with T2DM, and 17 were nondiabetic matched CTRL subjects. Among these 32 T2DM subjects, 13 subjects received neither metformin nor insulin treatment (no antidiabetic medications [*n* = 8] or other oral antidiabetic medications [*n* = 5]), 5 subjects received metformin but no insulin treatment before their death, 8 subjects were under insulin treatment without additional metformin treatment, and 6 subjects received both metformin and insulin treatment (Supplemental Figure 2). The duration of T2DM could not be determined for each individual, owing to the fact that the start of diabetic pathology could be far earlier than the diagnosis and start of treatment.

T2DM and CTRL groups were matched for sex, age, BMI, brain weight, time of death, month of death, fixation time, and Braak stage, but differed in PMD (Table 1). Table 1 shows the most recent data, although incomplete, on postabsorptive blood glucose and HbA1c, a measure of 3-month average glucose levels. The T2DM subgroups, when separated according to metformin or insulin treatment, matched for all the previously mentioned confounding factors (Table 1). An overview of more details of each individual subject, medication use, clinical diagnosis, and cause of death is provided in Supplemental Table 1.

### Histology.

After autopsy, the isolated hypothalamic tissues were immediately immersed in formalin and fixed at room temperature 1–2 months (Supplemental Table 1). Tissues were then ethanol-dehydrated, toluene-cleared, and paraffin-embedded. Sequential hypothalamic sections (6 μm) were sectioned along the rostro-caudal axis, from the lamina terminalis to the mammillary bodies. The anatomical orientation and rostro-caudal range of the IFN was first determined by Nissl staining, and subsequently precisely established by NPY-ir of every 100th section (Figure 1). For every subject, the section with the highest number of NPY-ir soma was determined, and all the following stainings were performed on 2 sections in close proximity to this section.

For both procedures, sections were mounted on glass slides (superfrost+) and dried on a 37°C heating plate. After 48 hours, sections were deparaffinized in 100% xylene, rehydrated in grading ethanol (100%–50%), and rinsed in distilled water. The immunohistochemical procedure of the NPY staining is similar to that of the other stainings and described below.

### Immunohistochemistry.

Heat-induced epitope retrieval was performed in citrate buffer, pH 6.0, using microwave treatment (10 minutes at 700 W) before incubation with POMC, Iba1, and TMEM119 (NPY does not require antigen retrieval). After cooling, sections were treated with 3% H_2_O_2_ in TBS (0.05 M Tris, 0.15 M NaCl, pH 7.6) for 10 minutes, washed in TBS, and incubated with the primary antibody overnight at 4°C. The primary antibodies against POMC and NPY were diluted in SUMI-milk (5% nonfat milk, 0.25% gelatin, 0.5% Triton X-100 in TBS), and those against iba1 and TMEM119 (i.e., 2 markers for microglia) were diluted in SUMI without milk. The next day, sections were washed in TBS, incubated for 60 minutes with biotinylated horse anti–rabbit IgG antibody (1:400, Vector Laboratories), washed in TBS, incubated with avidin–biotin complex (1:800, Vectastain Elite ABC kit; Vector Laboratories Inc.), and washed in TBS. Finally, sections were incubated in 0.5 mg/mL 3,3’-Diaminobenzidine (Sigma Chemical Co.) in TBS containing 0.2% ammonium nickel sulfate (BDH; Brunschwig) and 0.01% H_2_O_2_ (Merck) for 20 minutes. The reaction was stopped in distilled water. Next, sections were dehydrated with ethanol, cleared using xylene, and coverslipped with Entellan mounting medium. Primary antibodies used were rabbit polyclonal anti-POMC (1:1000, Phoenix Pharmaceuticals, 27-52), rabbit polyclonal anti-NPY (1:1000, Netherlands Institute for Brain Research; Niepke 261188) (47), rabbit polyclonal anti-iba1 (1:200, Synaptic Systems, 234003), and rabbit monoclonal anti-TMEM119 (1:400, Abcam, ab209064). The specificities of these primary antibodies were confirmed by comparison with negative control staining.

### Quantitative analysis.

Quantitative image analysis was performed by a researcher blind to the clinical information of the subjects. Neuronal and microglial cell numbers were counted using Fiji, an ImageJ distribution (48). Quantification of POMC- and NPY-expressing neurons was performed by manually outlining the area of interest based on the location of positive signal. Subsequently, using the “particle analysis” tool, the number and size of all immunoreactive positive particles in the outline were calculated, and particles larger than 30 μm^2^ were considered a neuronal soma. This size was determined in a pilot study for both NPY-ir and POMC-ir neurons, measuring the average size of the smallest nucleolus-containing neurons. Total soma number was divided by the area of the outline, resulting in soma number/mm^2^. The size of all somata was averaged and resulted in average soma size (μm^2^). Finally, the percentage of outlined area occupied by total immunoreactive positive particles was calculated as percent area masked.

For microglia visualized by iba1-ir and TMEM119-ir, the number of positive cells was calculated in the outlines generated by the corresponding POMC and NPY stainings (Supplemental Figure 1, C and D). Positive particles larger than 20 μm^2^ and smaller than 100 μm^2^ (size determined in pilot study) were considered a microglial soma, resulting in soma number/mm^2^. Soma size of all somas was averaged, resulting in average soma size (μm^2^). The number of particles between 6 μm^2^ and 20 μm^2^ were counted as number of ramifications. Total number of ramifications was divided by area of outline, resulting in ramification number/mm^2^.

### Statistics.

We performed a D’Agostino and Pearson normality test to determine if data were normally distributed. Normal distributed data were further analyzed using a 2-tailed Student’s *t* test. Data not following a normal distribution were further analyzed using a Mann-Whitney test. We controlled for multiple testing using the Benjamini-Hochberg criterion (49), resulting in an adjusted *P* value (q-value). Treatment-associated effects were analyzed using 2-way ANOVA. Correlations were measured using linear regression. A *P* value smaller than 0.05 was considered statistically significant. All statistical tests were performed using GraphPad Prism 8.12.

### Study approval.

All brain material was obtained from the Netherlands Brain Bank. The donor or next of kin gave informed consent for a brain autopsy and for the use of the brain material and medical records for research purposes.

## Author contributions

CXY designed the overall research. MJTK, SECW, and NLK performed immunohistochemical studies and data analysis. SELF, JAR, EF, AK, DFS, IH, and EMH critically reviewed the manuscript. CXY and MJTK conceived and designed experiments, analyzed the data, and wrote the manuscript. CXY supervised the project. All authors approved the final version of the manuscript.

## Supplementary Material

Supplemental data

## Figures and Tables

**Table 1:**
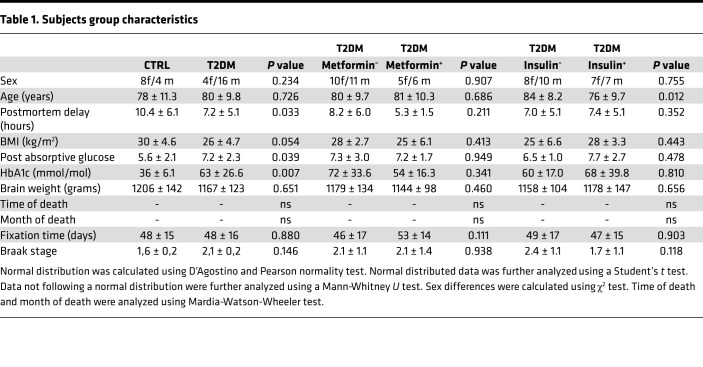
Subjects group characteristics

## Version 1 08/20/2020

Electronic publication
