# Supplementary material for: The impact of antidiabetic treatment on human hypothalamic infundibular neurons and microglia
Source: JCI Insight. 2020 Aug 20;5(16):e133868. doi: 10.1172/jci.insight.133868 (PMC7455135; doi:10.1172/jci.insight.133868)
Supplement: Supplemental data [file jciinsight-5-133868-s225.pdf]

Supplemental figures and figure legends

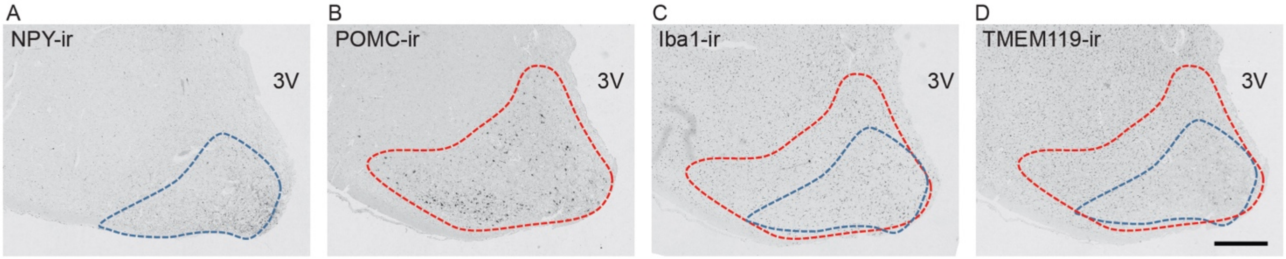

Supplemental Figure 1. Outlines of immunoreactivity of neurons and microglia in the IFN. (**A**) The neuropeptide-Y immunoreactive (NPY-ir) area is framed by a blue line in the infundibular nucleus (IFN) for data analysis; (**B**) The pro-opiomelanocortin immunoreactive (POMC-ir) area is framed by a red line in the IFN for data analysis; (**C**) The ionized calcium-binding adapter molecule 1 immunoreactive (iba1-ir) microglia in the IFN were separately analyzed in both the areas framed by the blue and the red lines; (**D**) The transmembrane protein 119 immunoreactive (TMEM119-ir) microglia were separately analyzed in both the areas framed by the blue and the red lines. Panel A is cropped from Figure 2A, panel B from Figure 3A, panel C from Figure 4A and panel D from Figure 5A. 3V: third ventricle. Scale bar: 500 µm in D.

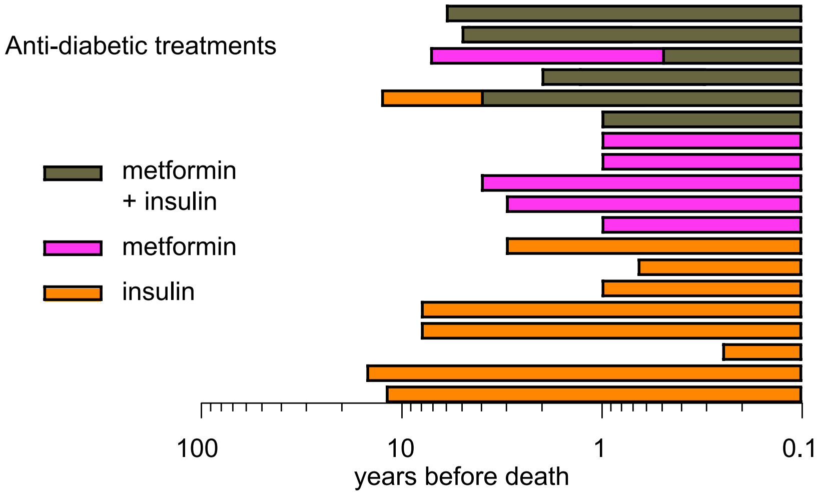

Supplemental Figure 2. An overview of the treatment history of the T2DM subjects which received either metformin and/or insulin treatment. Metformin is shown in pink, insulin in orange and the combination is shown in olive green.

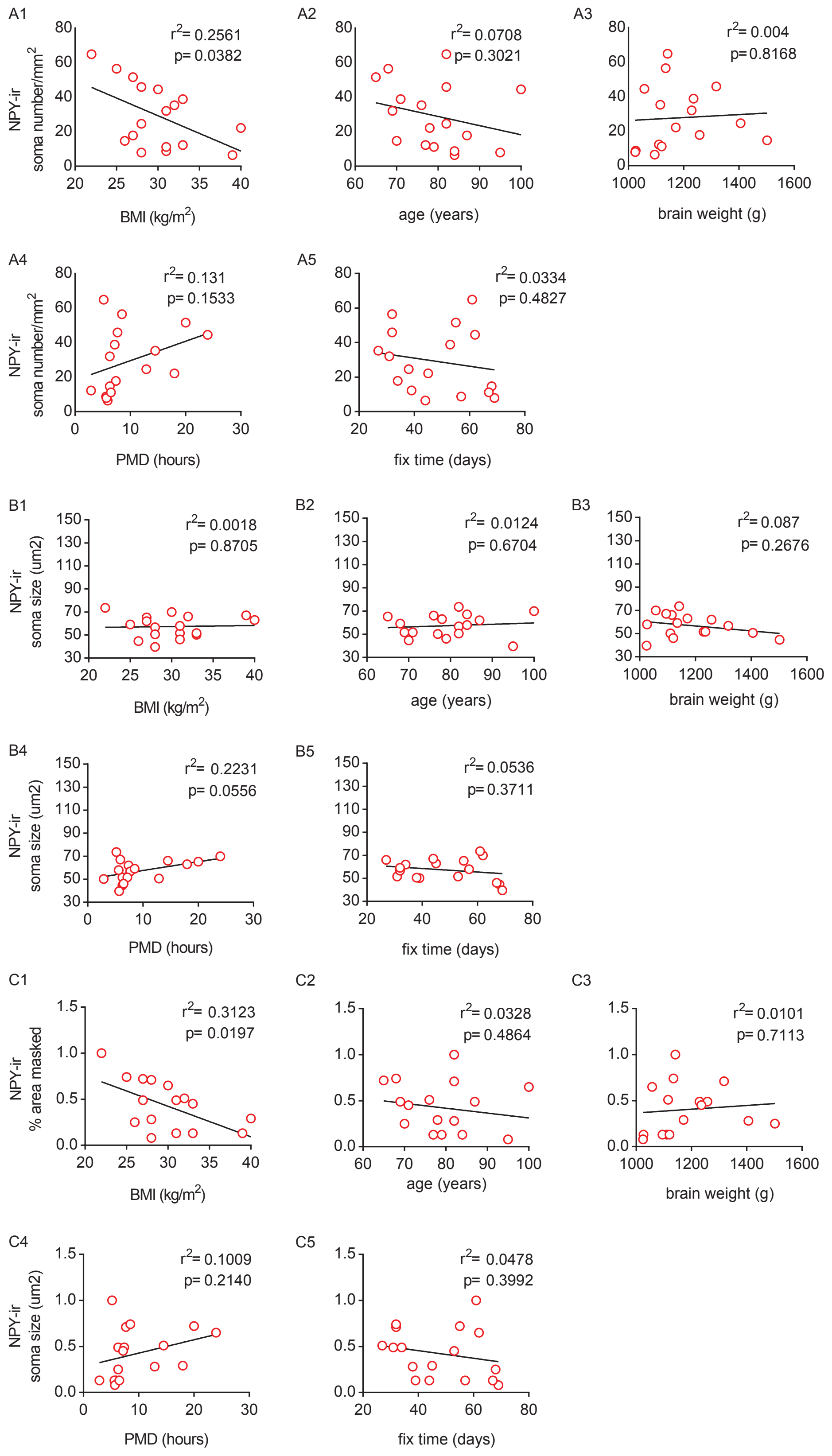

Supplemental Figure 3. Confounders analysis with NPY-ir in CTRL subjects. (**A**) Plots of the number of neuropeptide-Y immunoreactive (NPY-ir) neurons in CTRL subjects according to body mass index (BMI) (A1), age (A2), brain weight (A3), post-mortem delay (PMD) (A4) and fixation time (A5). (**B**) Plots of average soma size of NPY-ir neurons in CTRL subjects according to BMI (B1), age (B2), brain weight (B3), PMD (B4) and fixation time (B5). (**C**) Plots of relative area covered by NPY-ir neurons in CTRL subjects according to BMI (C1), age (C2), brain weight (C3), PMD (C4) and fixation time (C5).

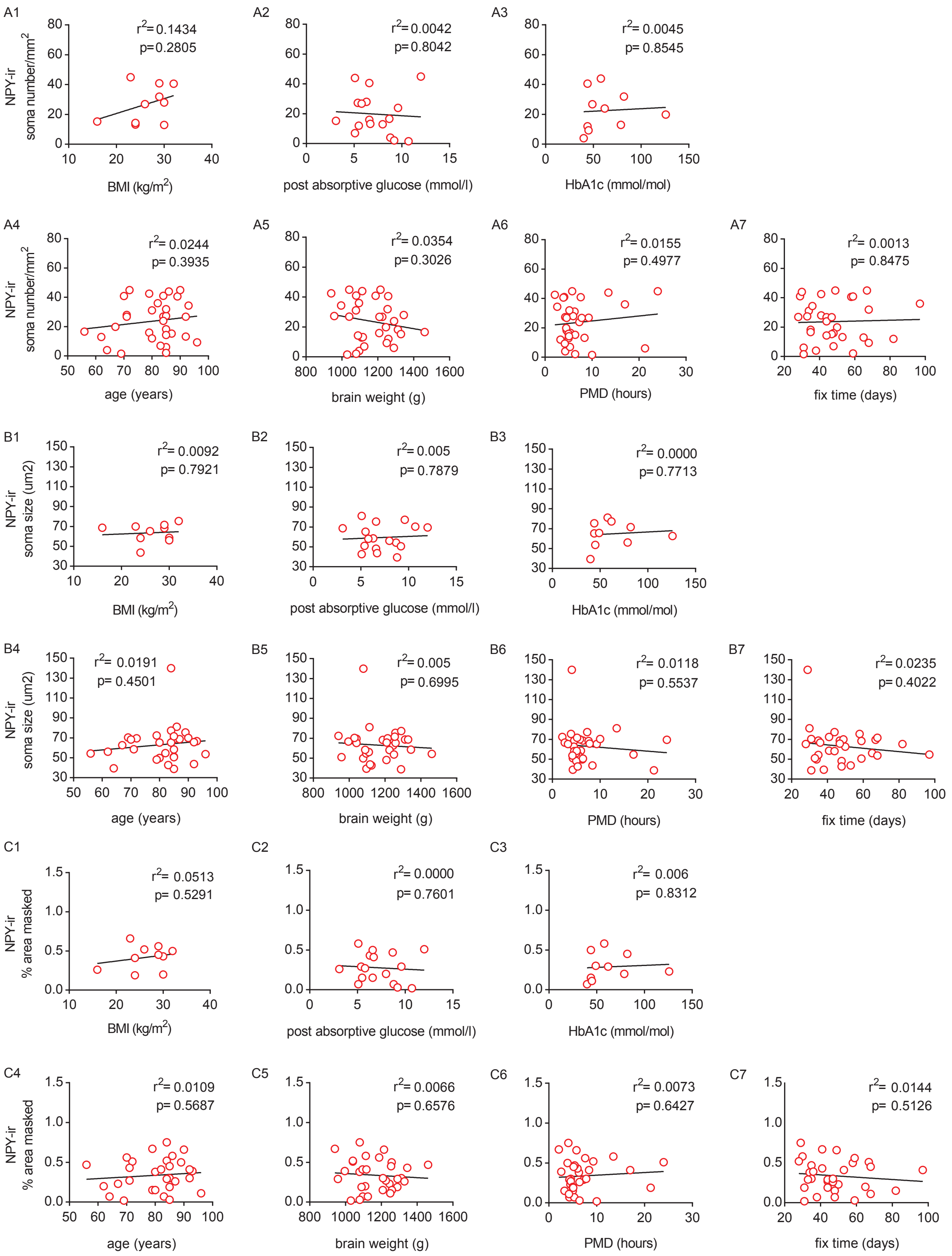

Supplemental Figure 4. Confounders analysis with NPY-ir in T2DM subjects. (A) Plots of the number of neuropeptide-Y immunoreactive (NPY-ir) neurons in T2DM subjects according to body mass index (BMI) (A1), post absorptive glucose (A2), HbA1c (A3), age (A4), brain weight (A5), post-mortem delay (PMD) (A6) and fixation time (A7). (**B**) Plots of average soma size of NPY -ir neurons in T2DM subjects according to BMI (B1), post absorptive glucose (B2), HbA1c (B3), age (B4), brain weight (B5), PMD (B6) and fixation time (B7). (**C**) Plots of relative area covered by NPY -ir neurons in T2DM subjects according to BMI (C1), post absorptive glucose (C2), HbA1c (C3), age (C4), brain weight (C5), PMD (C6) and fixation time (C7).

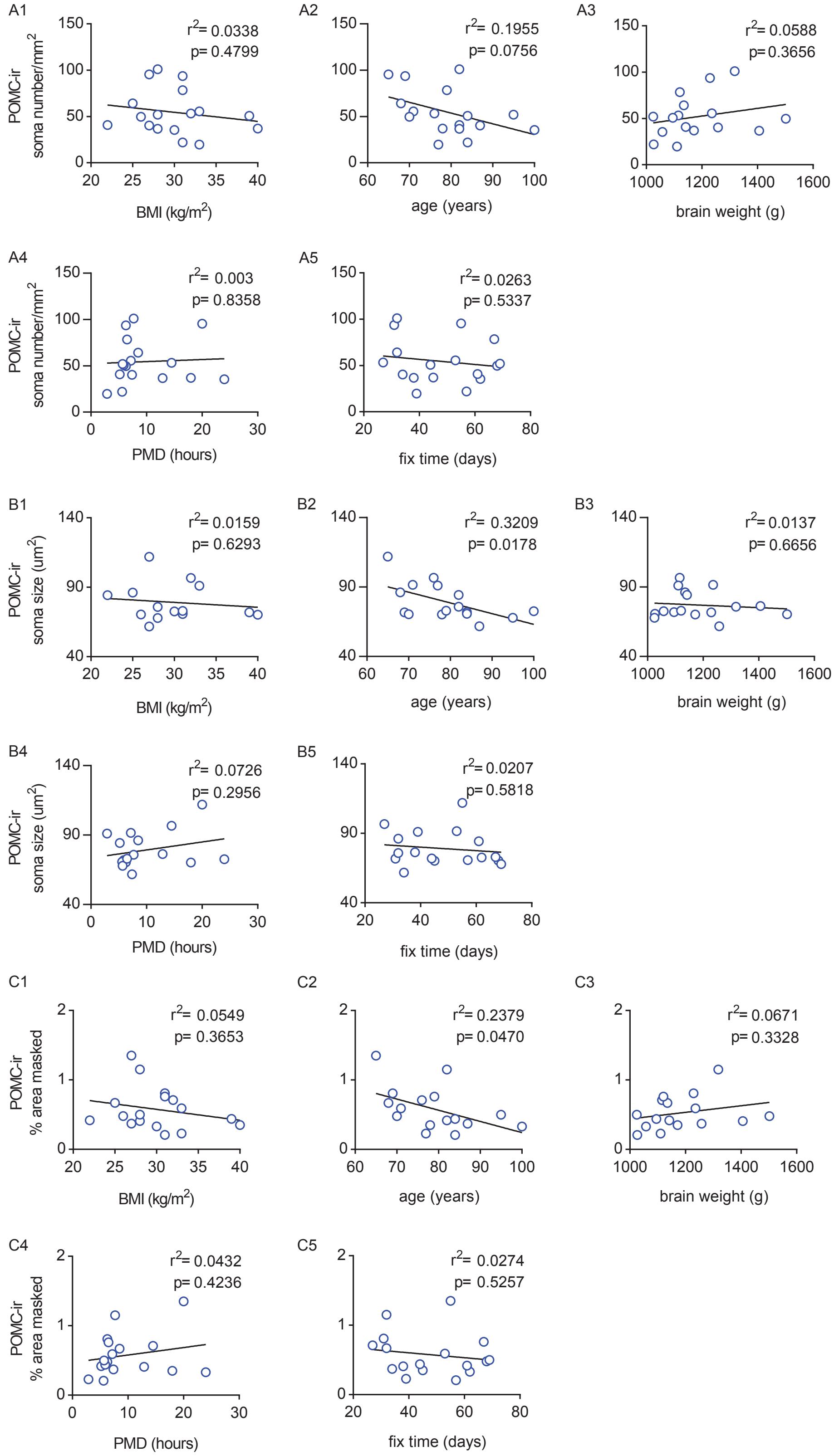

Supplemental Figure 5. Confounders analysis with POMC-ir in CTRL subjects. (**A**) Plots of the number of pro-opiomelanocortin immunoreactive (POMC-ir) neurons in CTRL subjects according to body mass index (BMI) (A1), age (A2), brain weight (A3), post-mortem delay (PMD) (A4) and fixation time (A5). (**B**) Plots of average soma size of POMC-ir neurons in CTRL subjects according to BMI (B1), age (B2), brain weight (B3), PMD (B4) and fixation time (B5). (**C**) Plots of relative area covered by POMC-ir neurons in CTRL subjects according to BMI (C1), age (C2), brain weight (C3), PMD (C4) and fixation time (C5).

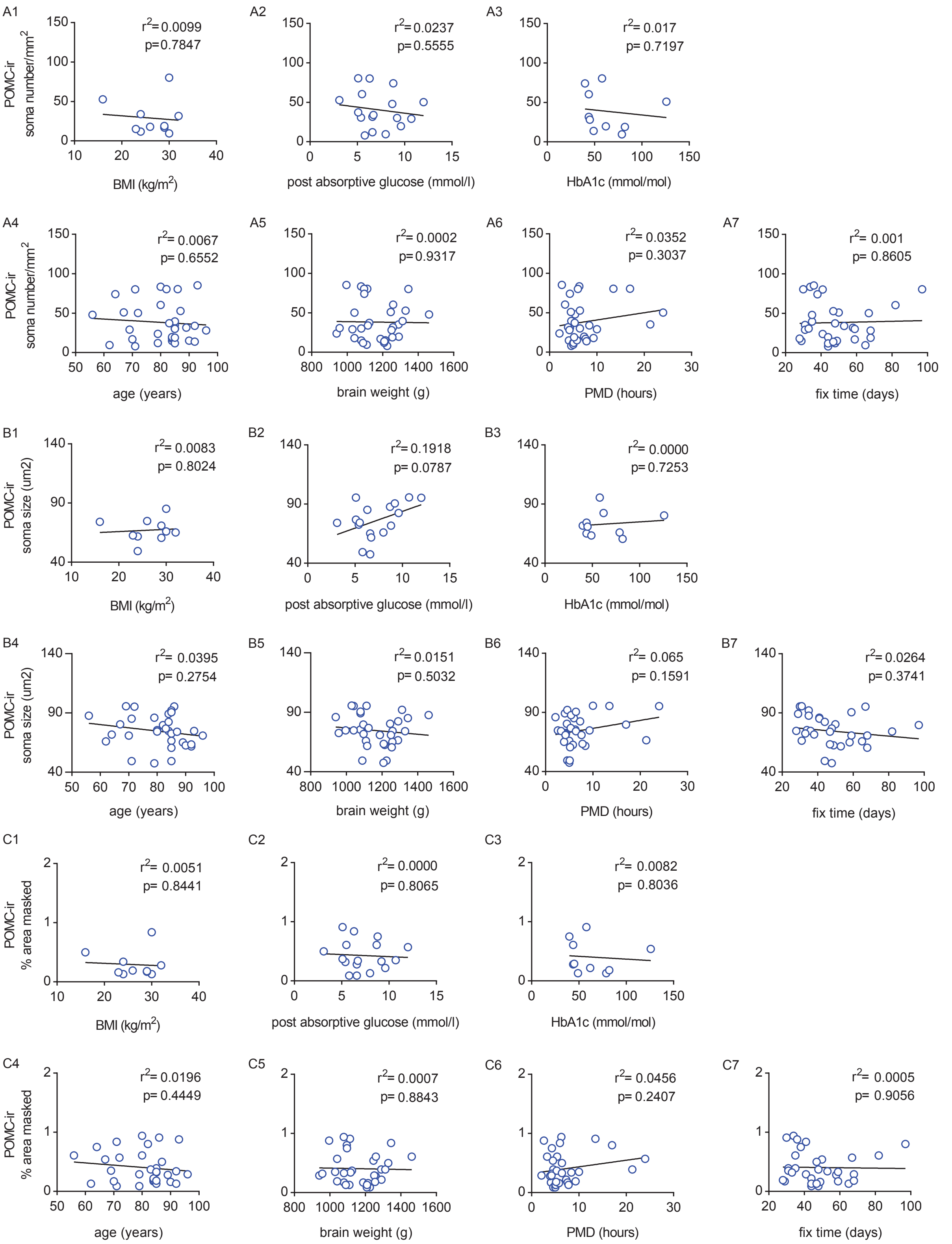

Supplemental Figure 6. Confounders analysis with POMC-ir in T2DM subjects. (**A**) Plots of the number of pro-opiomelanocortin immunoreactive (POMC-ir) neurons in T2DM subjects according to body mass index (BMI) (A1), post absorptive glucose (A2), HbA1c (A3), age (A4), brain weight (A5), post-mortem delay (PMD) (A6) and fixation time (A7). (**B**) Plots of average soma size of POMC-ir neurons in T2DM subjects according to BMI (B1), post absorptive glucose (B2), HbA1c (B3), age (B4), brain weight (B5), PMD (B6) and fixation time (B7). (**C**) Plots of relative area covered by POMC-ir neurons in T2DM subjects according to BMI (C1), post absorptive glucose (C2), HbA1c (C3), age (C4), brain weight (C5), PMD (C6) and fixation time (C7).

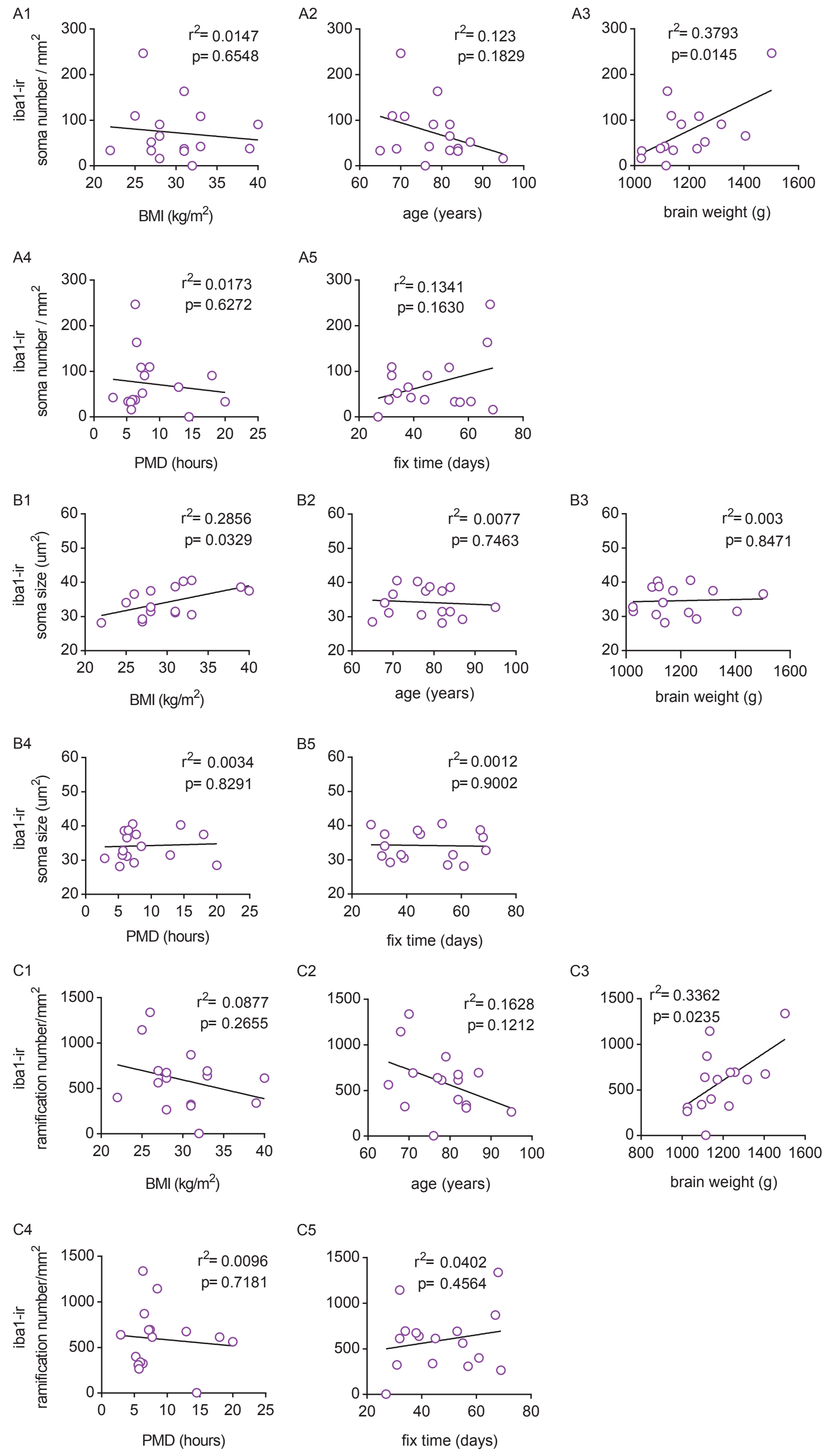

Supplemental Figure 7. Confounders analysis with iba1-ir in NPY region of CTRL subjects. (**A**) Plots of the number of ionized calcium-binding adapter molecule 1 immunoreactive (iba1-ir) microglia in the neuropeptide Y (NPY) region of CTRL subjects according to body mass index (BMI) (A1), age (A2), brain weight (A3), post-mortem delay (PMD) (A4) and fixation time (A5). (**B**) Plots of average soma size of iba1-ir microglia in the NPY region of CTRL subjects according to BMI (B1), age (B2), brain weight (B3), PMD (B4) and fixation time (B5). (**C**) Plots of the number of iba1-ir microglial ramifications in the NPY region of CTRL subjects according to BMI (C1), age (C2), brain weight (C3), PMD (C4) and fixation time (C5).

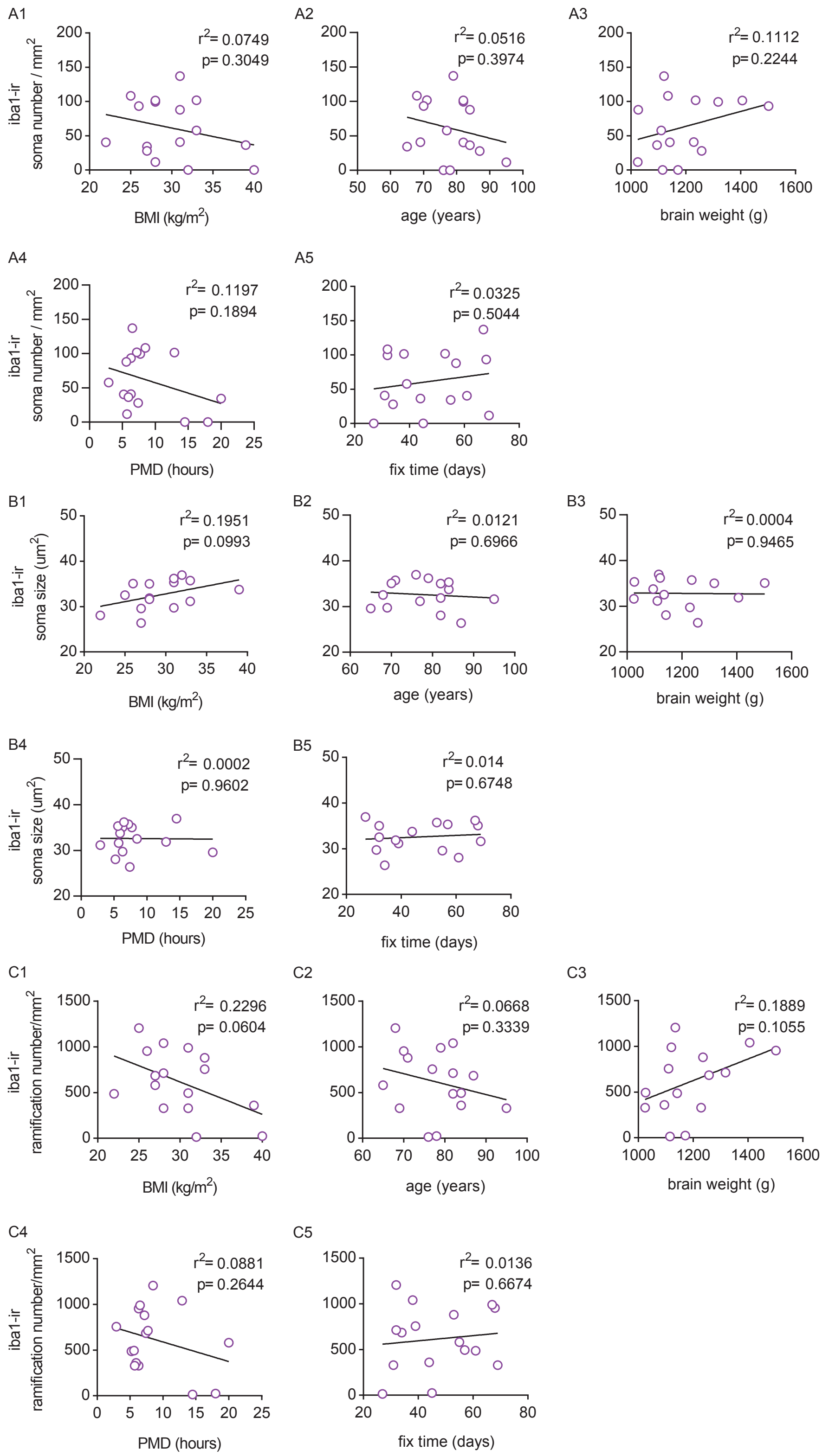

Supplemental Figure 8. Confounders analysis with iba1-ir in POMC region of CTRL subjects. (A) Plots of the number of ionized calcium-binding adapter molecule 1 immunoreactive (iba1-ir) microglia in the pro-opiomelanocortin (POMC) region of CTRL subjects according to body mass index (BMI) (A1), age (A2), brain weight (A3), post-mortem delay (PMD) (A4) and fixation time (A5). (**B**) Plots of average soma size of iba1-ir microglia in the pro-opiomelanocortin (POMC) region of CTRL subjects according to BMI (B1), age (B2), brain weight (B3), PMD (B4) and fixation time (B5). (**C**) Plots of the number of iba1-ir microglial ramifications in the POMC region of CTRL subjects according to BMI (C1), age (C2), brain weight (C3), PMD (C4) and fixation time (C5).

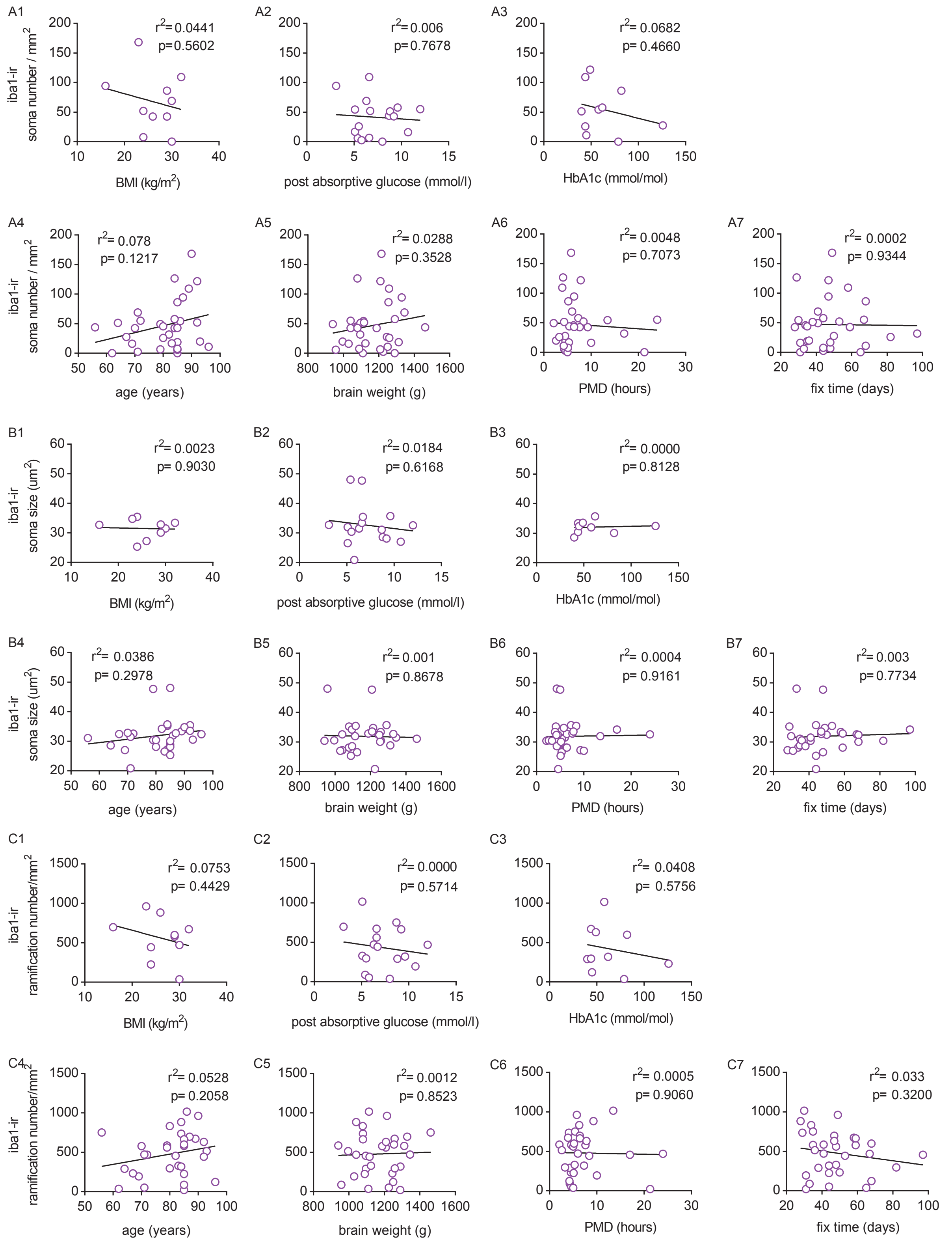

Supplemental Figure 9. Confounders analysis with iba1-ir in NPY region of T2DM subjects. (A) Plots of the number of ionized calcium-binding adapter molecule 1 immunoreactive (iba1-ir) microglia in the neuropeptide Y (NPY) region of T2DM subjects according to body mass index (BMI) (A1), post absorptive glucose (A2), HbA1c (A3), age (A4), brain weight (A5), post-mortem delay (PMD) (A6) and fixation time (A7). (**B**) Plots of average soma size of iba1-ir microglia in the NPY region of T2DM subjects according to BMI (B1), post absorptive glucose (B2), HbA1c (B3), age (B4), brain weight (B5), PMD (B6) and fixation time (B7). (**C**) Plots of iba1-ir microglial ramifications in the NPY region of T2DM subjects according to BMI (C1), post absorptive glucose (C2), HbA1c (C3), age (C4), brain weight (C5), PMD (C6) and fixation time (C7).

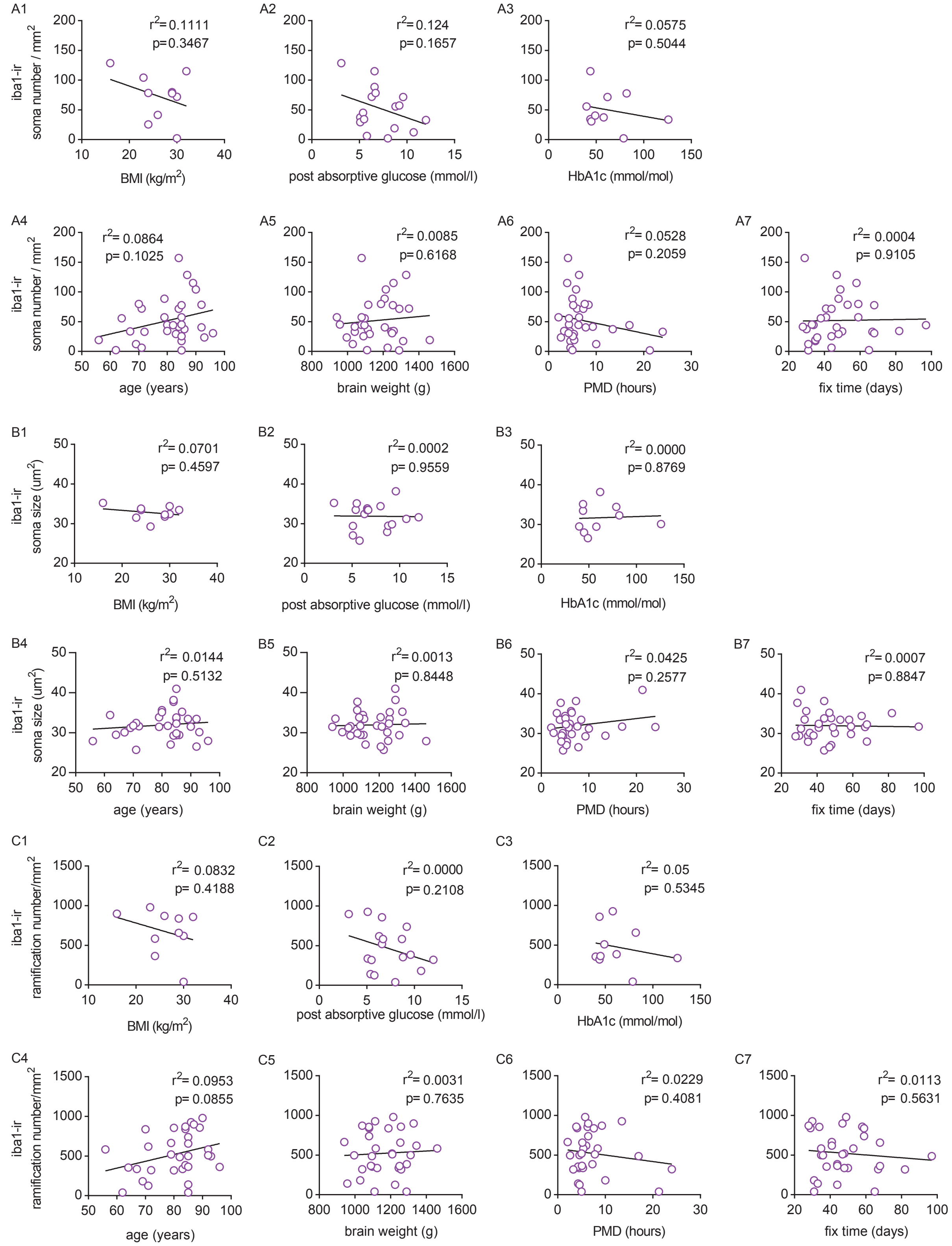

Supplemental Figure 10. Confounders analysis with iba1-ir in POMC region of T2DM subjects. (**A**) Plots of the number of ionized calcium-binding adapter molecule 1 immunoreactive (iba1-ir) microglia in the pro-opiomelanocortin (POMC) region of T2DM subjects according to body mass index (BMI) (A1), post absorptive glucose (A2), HbA1c (A3), age (A4), brain weight (A5), post-mortem delay (PMD) (A6) and fixation time (A7). (**B**) Plots of average soma size of iba1-ir microglia in the POMC region of T2DM subjects according to BMI (B1), post absorptive glucose (B2), HbA1c (B3), age (B4), brain weight (B5), PMD (B6) and fixation time (B7). (**C**) Plots of iba1-ir microglial ramifications in the POMC region of T2DM subjects according to BMI (C1), post absorptive glucose (C2), HbA1c (C3), age (C4), brain weight (C5), PMD (C6) and fixation time (C7).

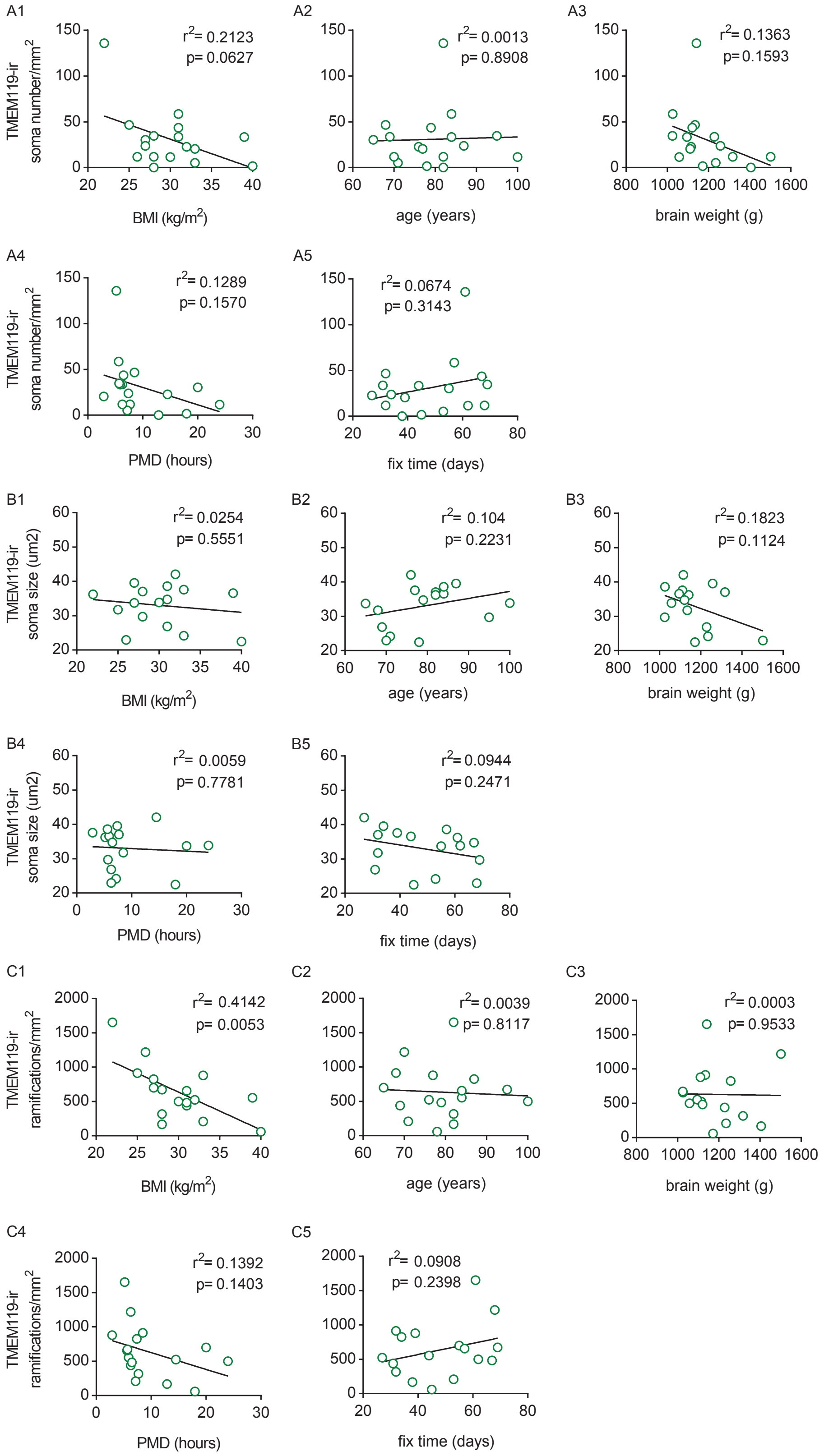

Supplemental Figure 11. Confounders analysis with TMEM-ir in NPY region of CTRL subjects. (A) Plots of the number of transmembrane protein 119 immunoreactive (TMEM119-ir) microglia in the neuropeptide Y (NPY) region of CTRL subjects according to body mass index (BMI) (A1), age (A2), brain weight (A3), post-mortem delay (PMD) (A4) and fixation time (A5). (**B**) Plots of average soma size of TMEM119-ir microglia in the NPY region of CTRL subjects according to BMI (B1), age (B2), brain weight (B3), PMD (B4) and fixation time (B5). (**C**) Plots of the number of TMEM119-ir microglial ramifications in the NPY region of CTRL subjects according to BMI (C1), age (C2), brain weight (C3), PMD (C4) and fixation time (C5).

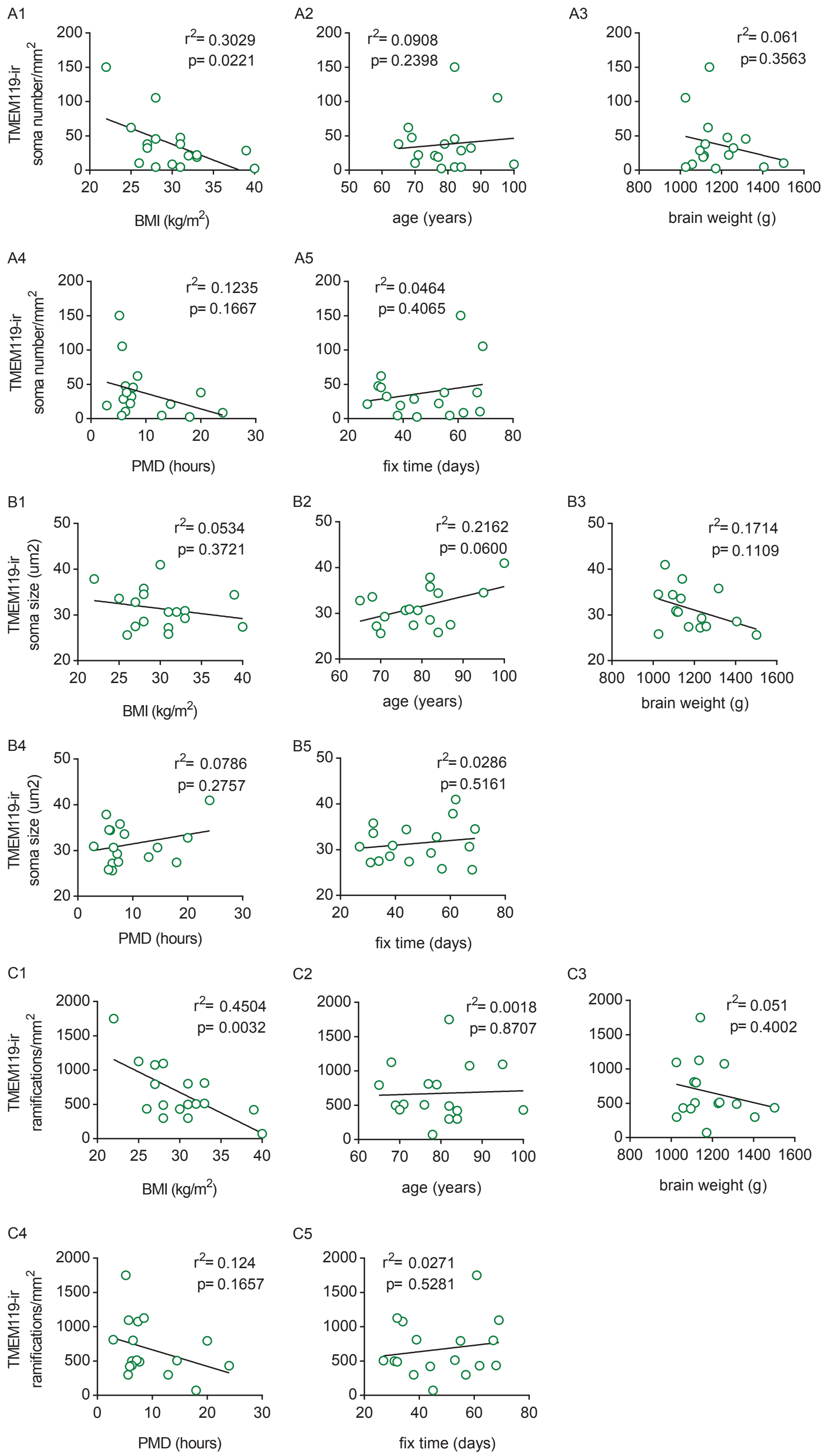

Supplemental Figure 12. Confounders analysis with TMEM119-ir in POMC region of CTRL subjects. (**A**) Plots of the number of transmembrane protein 119 immunoreactive (TMEM119-ir) microglia in the pro-opiomelanocortin (POMC) region of CTRL subjects according to body mass index (BMI) (A1), age (A2), brain weight (A3), post-mortem delay (PMD) (A4) and fixation time (A5). (**B**) Plots of average soma size of TMEM119-ir microglia in the POMC region of CTRL subjects according to BMI (B1), age (B2), brain weight (B3), PMD (B4) and fixation time (B5). (**C**) Plots of the number of TMEM119-ir microglial ramifications in the POMC region of CTRL subjects according to BMI (C1), age (C2), brain weight (C3), PMD (C4) and fixation time (C5).

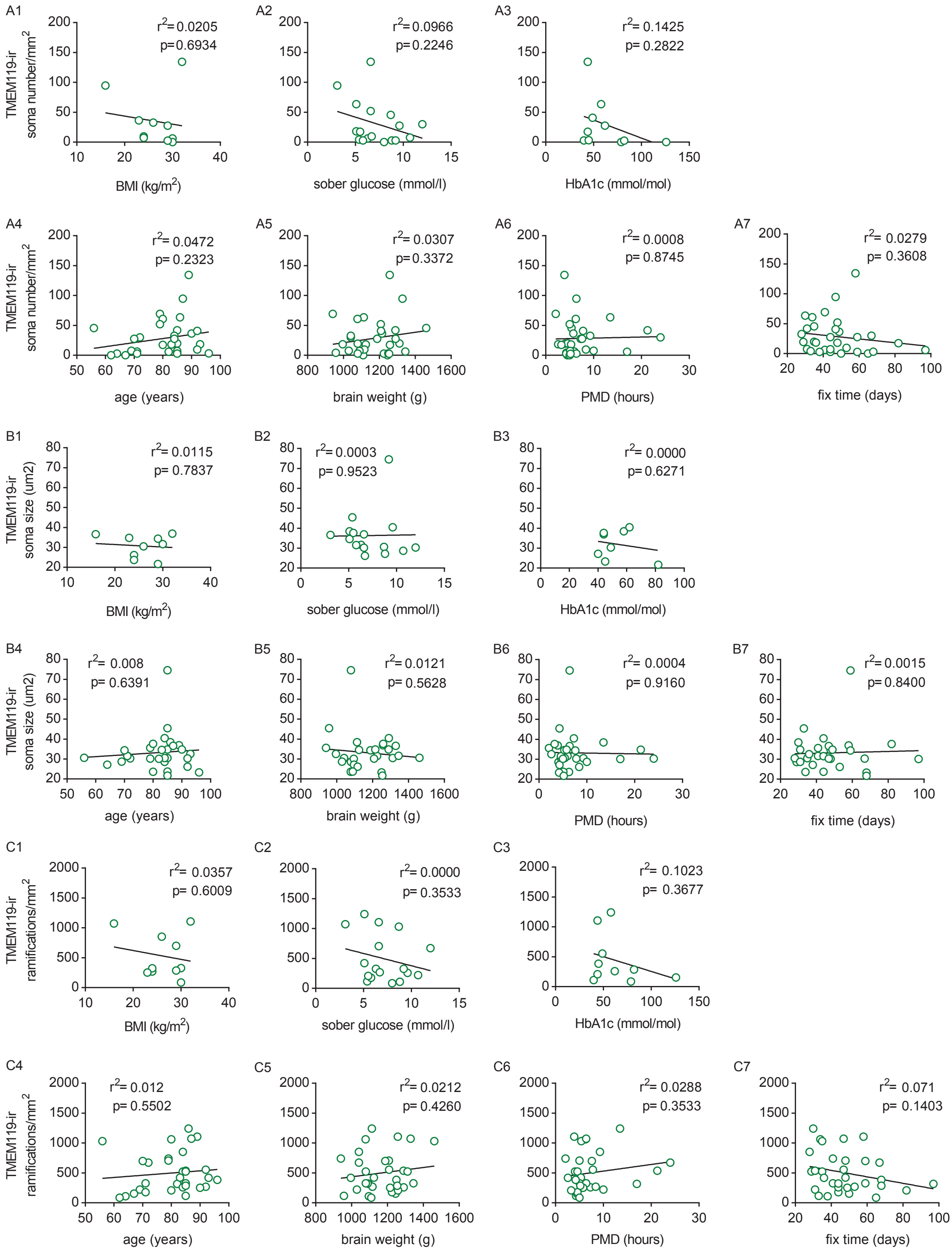

Supplemental Figure 13. Confounders analysis with TMEM119-ir microglia in NPY region of T2DM subjects. (A) Plots of the number of transmembrane protein 119 immunoreactive (TMEM119-ir) microglia in the neuropeptide Y (NPY) region of T2DM subjects according to body mass index (BMI) (A1), post absorptive glucose (A2), HbA1c (A3), age (A4), brain weight (A5), post-mortem delay (PMD) (A6) and fixation time (A7). (**B**) Plots of average soma size of TMEM119-ir microglia in the NPY region of T2DM subjects according to BMI (B1), post absorptive glucose (B2), HbA1c (B3), age (B4), brain weight (B5), PMD (B6) and fixation time (B7). (**C**) Plots of TMEM119-ir microglial ramifications in the NPY region of T2DM subjects according to BMI (C1), post absorptive glucose (C2), HbA1c (C3), age (C4), brain weight (C5), PMD (C6) and fixation time (C7).

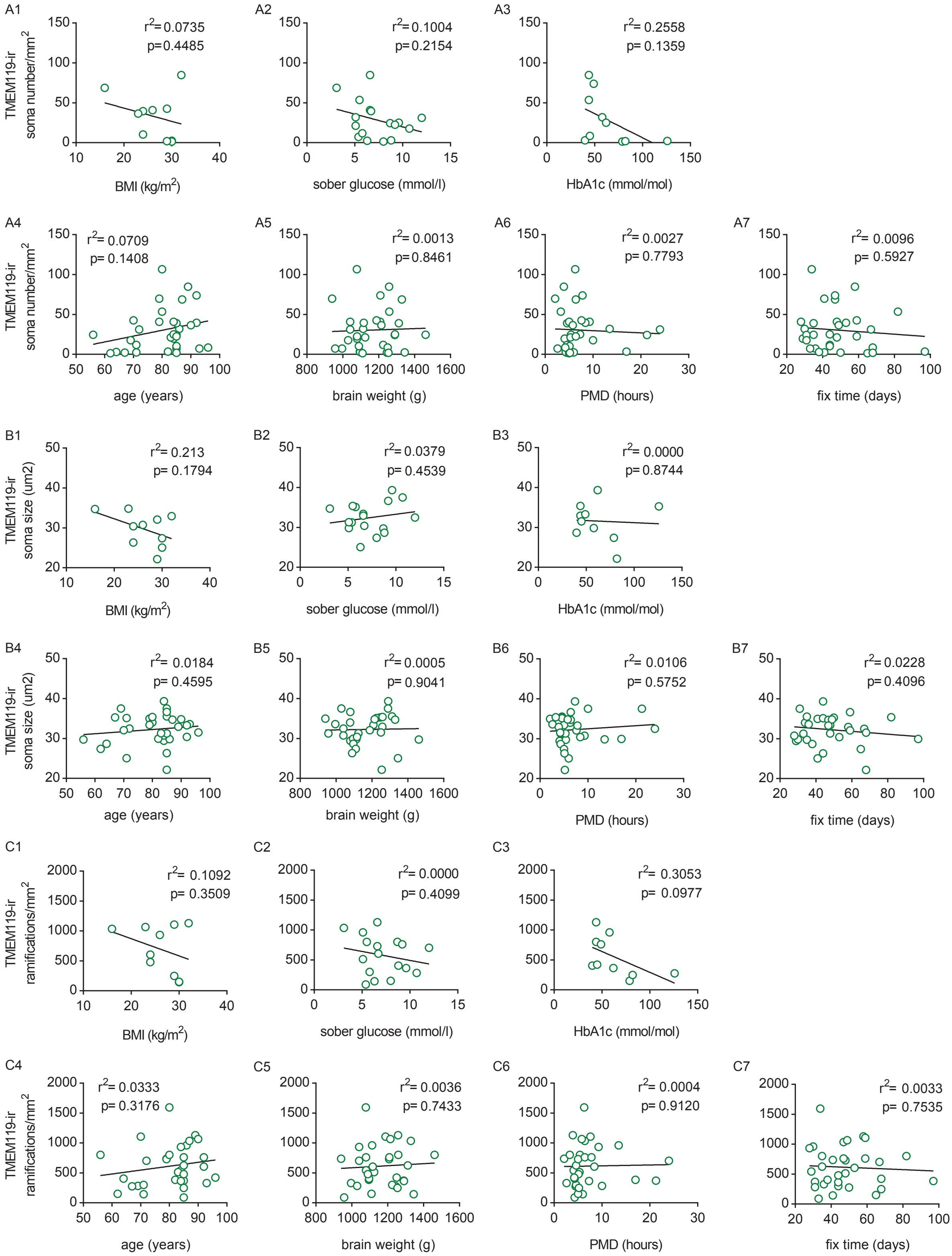

Supplemental Figure 14. Confounders analysis with TMEM119-ir microglia in POMC region of T2DM subjects. (**A**) Plots of the number of transmembrane protein 119 immunoreactive (TMEM119-ir) microglia in the pro-opiomelanocortin (POMC) region of T2DM subjects according to body mass index (BMI) (A1), post absorptive glucose (A2), HbA1c (A3), age (A4), brain weight (A5), post-mortem delay (PMD) (A6) and fixation time (A7). (**B**) Plots of average soma size of TMEM119-ir microglia in the POMC region of T2DM subjects according to BMI (B1), post absorptive glucose (B2), HbA1c (B3), age (B4), brain weight (B5), PMD (B6) and fixation time (B7). (**C**) Plots of TMEM119-ir microglial ramifications in the POMC region of T2DM subjects according to BMI (C1), post absorptive glucose (C2), HbA1c (C3), age (C4), brain weight (C5), PMD (C6) and fixation time (C7).

Supplementary Table 1. Clinico-pathological data of patients and matched controls.

NBB	sex	age	PMD	BMI	post-abs. glucose	HbA1c	fixation time	Braak stage	Metformin	Insulin	Cause of death and clinical diagnosis
**Control**											
1997-065	f	76	14.5	32	/	/	27	/	no	no	Myocardial infarction, unilateral nephrectomy and adrenalectomy.
1997-146	f	100	24.0	30	4.4	/	62	2	no	no	Pneumonia, myocardial infarction, dyspnea.
1998-035	f	65	20.0	27	/	/	55	0	no	no	Mesenterial ischemia complications, dyspnea, atrial fibrillation.
1998-036	f	69	6.3	31	/	/	31	1	no	no	Cardiac shock, cholecystolithiasis, hypothyroidism.
1998-049	m	87	7.4	27	/	/	34	2	no	no	Cardiac arrest.
2000-072	m	78	18.0	40	1.4	/	45	1	no	no	Kidney failure, dehydration, heart failure, renal insufficiency.
2001-021	m	82	7.7	28	/	/	32	1	no	no	Heart attack, ischemic heart disease, kyphosis of backbone.
2001-069	f	68	8.5	25	/	/	32	1	no	no	Legal euthanasia, vaginal carcinoma, kidney tumor and lung metastasis.
2007-088	f	82	5.2	22	4.3	/	61	3	no	no	Cachexia, cardiac failure, encephalopathy, mitral valve insufficiency.
2009-022	f	77	2.9	33	/	/	39	1	no	no	Pulmonary metastasis of vulva carcinoma.
2009-039	m	82	12.9	28	7.4	/	38	3	no	no	Heart failure, prostate carcinoma.
2009-095	f	71	7.2	33	/	31	53	1	no	no	Renal failure, CVA, hypertensive retinopathy.
2010-013	m	70	6.3	26	/	31	68	0	no	no	Acute myocardial infarction, prostate carcinoma.
2011-082	f	84	5.9	39	6.2	41	44	2	no	no	Respiratory failure, angina pectoris, mitral valve insufficiency.
2012-005	f	84	5.6	31	7.5	42	57	2	no	no	Heart failure, metastatic breast cancer, scoliosis.
2012-033	f	95	5.7	28	/	/	69	3	no	no	Heart failure, cachexia and dehydration, pulmonary disease.
2012-104	m	79	6.5	31	7.4	/	67	2	no	no	Legal euthanasia, ischemic colitis, heart failure with dyspnoea.
**T2DM**											
1989-032	m	84	4.1	/	/	/	29	/	no	no	Heart failure, intestinal tumour.
1995-008	f	79	2.1	/	/	/	41	3	no	no	Dehydration, endometrium carcinoma.
1995-016	f	86	13.5	/	5.1	58	30	2	no	no	Cardiac decompensation, refluxesophagitis.
1995-078	f	80	6.3	/	/	/	34	2	no	no	Dehydration, angina pectoris.
1998-055	m	85	21.3	/	/	/	31	/	no	no	Cardiac tamponade after a myocardial infarction.
1998-062	m	85	4.6	/	/	/	35	1	no	no	Respiratory insufficiency, metastasized adenocarcinoma.
1998-080	f	72	24.0	/	12	/	67	3	no	yes	Cardiac decompensation, complete respiratory insufficiency.
1998-112	f	84	9.3	/	/	/	28	3	no	yes	Pulmonary emboli, CVA, atherosclerosis.
1998-126	m	71	6.0	26	6.3	/	41	2	no	yes	Respiratory insufficiency, lung carcinoma.
1998-127	m	56	5.4	30	8.7	/	35	0	no	yes	Cardiac infarction, mitral valve insufficiency, ischaemic left CVA.
1998-150	f	82	17.0	/	/	/	97	0	no	no	Respiratory insufficiency, basal cell carninoma, right-sided nephrectomy.
1999-015	f	93	2.6	/	/	/	36	3	no	no	Pneumonia, dehydration, breast cancer.
2001-003	m	69	10.0	/	10.7	/	31	/	no	yes	Cardiac arrest, urinary tract infection and fever, cholelithiasis.
2001-061	f	85	4.3	/	5.4	/	33	2	yes	yes	Myocardial infarction, parkinsonism and depression.
2003-054	m	67	4.5	/	/	126	50	1	no	yes	Cardiac shock, CVA.
2004-085	f	71	4.6	/	5.8	/	44	3	no	no	Dehydration, CVA with right side paresis.
2005-027	f	64	4.3	/	8.8	40	38	0	yes	yes	Respiratory failure, CVA.
2006-033	m	79	5.0	/	6.6	/	48	1	yes	no	Pneumonia, dehydration, CVA, choledochus carcinoma.
2007-061	f	83	5.3	/	5.1	/	48	3	yes	yes	Cachexia, CVA, arteriosclerosis.
2008-061	f	62	5.0	/	8	79	65	1	yes	no	Cachexia, hyperthyroidism.
2008-105	f	89	3.9	30	6.6	44	58	3	no	no	Pneumonia, coronary artery bypass, atrial fibrillation.
2009-091	m	84	7.3	32	9.6	62	44	1	yes	yes	Anaemia, colon and prostate carcinoma metastasis.
2009-096	m	92	8.4	/	6.7	/	53	4	yes	no	Heart failure, myocardial infarction.
2009-104	m	87	6.4	24	3.1	/	47	1	no	yes	Kidney carcinoma, CVA.
2010-092	m	85	5.1	16	/	/	44	3	yes	no	Dehydration and cachexia, CVA.
2011-027	m	80	3.3	24	5.5	44	82	1	yes	yes	Pneumonia, CVA, ischemic attack.
2012-049	f	70	7.6	/	/	/	59	2	no	yes	Cachexia, pancreas carcinoma.
2012-088	f	85	6.4	29	9.2	/	59	3	yes	yes	Legal euthanasia, hypoparathyroidism.
2012-092	m	90	5.8	/	/	/	49	2	no	no	Prostate carcinoma, CVA.
2012-118	m	96	4.2	23	/	45	68	4	yes	no	Transient ischemic attack, urinary tract infection, diabetic retinopathy.
2014-040	m	85	5.2	/	/	82	68	3	no	no	Pulmonary carcinoma, pneumonia, arteritis temporalis.
2014-051	m	92	7.8	29	/	49	47	3	no	no	Liver cirrhosis ascites and anuria, hepatic cirrhosis.

NBB: Netherlands Brain Bank number; PMD: post-mortem delay (hours); post-abs. glucose: post-absorptive glucose; BMI: body mass index; HbA1C: glycated hemoglobin; CVA: cerebrovascular accident.
